# Recent Advances in the Hydrogen Gas Barrier Performance of Polymer Liners and Composites for Type IV Hydrogen Storage Tanks: Fabrication, Properties, and Molecular Modeling

**DOI:** 10.3390/polym17091231

**Published:** 2025-04-30

**Authors:** Omar Dagdag, Hansang Kim

**Affiliations:** Department of Mechanical Engineering, Gachon University, Seongnam 13120, Republic of Korea; omardagdag@gachon.ac.kr

**Keywords:** hydrogen barrier, polymer liners, type IV tanks, composites, hydrogen permeability, molecular modeling, high-pressure storage

## Abstract

Developing high-performance polymer liners and their composites is essential for ensuring the safety and efficiency of type IV high-pressure hydrogen storage tanks. This review provides a thorough analysis of recent innovations in hydrogen gas barrier materials, fabrication techniques, and molecular modeling approaches to minimize hydrogen gas permeation. It examines key polymeric materials, such as polyamide 6 (PA6) and high-density polyethylene (HDPE), and emerging nanofiller reinforcements, such as graphene and montmorillonite clay. Additionally, it discusses manufacturing methods in relation to their effects on liner integrity and permeability. Molecular modeling techniques, especially molecular dynamics simulations, are emphasized as powerful tools for understanding hydrogen transport mechanisms and optimizing the interactions between polymers and fillers. Despite these notable advancements, challenges remain in achieving ultra-low hydrogen gas permeability, long-term stability, and scalable production methods. Future research should focus on developing multifunctional hybrid fillers, enhancing computational modeling frameworks, and designing novel polymer architectures specifically tailored for hydrogen storage applications.

## 1. Introduction

Recently, interest in hydrogen fuel cell electric vehicles (FCEVs) has increased owing to their environmentally friendly characteristics. The utilization of hydrogen energy systems in vehicles has been suggested as a strategy for reducing greenhouse gas and other harmful pollutant emissions [1]. Type IV hydrogen tanks, which consist of carbon fiber-reinforced plastics and polymeric liners, offer promising eco-friendly advantages owing to the benefits associated with polymer composites [2]. Liners play a vital role in FCEVs because they prevent the escape of hydrogen gas from the tanks. Polymer composites are favored as hydrogen tank liner materials over heavy metals because of their lightweight nature and high impact strengths [3]. Moreover, these composites can integrate the beneficial properties of different polymers, leading to an enhanced overall performance, particularly in terms of mechanical strength, making them suitable for a variety of applications, including hydrogen tank liners. Despite the numerous benefits of polymeric materials, their high gas permeability significantly limits their use [4]. Consequently, extensive research has focused on developing polymer composites with reduced gas permeabilities to address the critical challenge of low gas barrier properties in hydrogen storage tanks. Researchers have investigated various approaches, including the incorporation of inorganic fillers, blending of crystalline polymers, and the application of chemical dissimilarity to decrease gas permeability in these materials [5].

The gas barrier mechanism of polymeric materials is primarily influenced by two factors. First, when gas molecules dissolve within a polymer, the chemical compatibility between the polymer and gas can increase the permeability, which can be quantified using the solubility coefficient. Specifically, the permeability coefficient incorporates the diffusion and solubility coefficients, with gas molecules diffusing through the polymer matrix driven by a concentration gradient. Second, the integration of fillers with high aspect ratios into polymeric materials creates tortuous pathways for gas diffusion. Longer and more intricate diffusion paths lead to a reduction in gas permeability as the diffusion process becomes more time-consuming [6]. This strategy of creating complex pathways is often preferred over simply relying on chemical similarity, as it yields significant improvements. Inorganic fillers such as montmorillonite clay and graphene oxide are frequently utilized to bolster the gas barrier properties of polymer composites because of their two-dimensional structures [7]. However, although these inorganic fillers enhance gas barrier properties, they may negatively affect the elongation characteristics of the polymers, which can restrict their applicability. Given the small size of H_2_ molecules, creating composites with substantial hydrogen gas barrier capabilities using only polymer materials is challenging. Consequently, additional research is necessary to develop effective liner materials.

To address these challenges, recent studies have focused on improving the hydrogen gas barrier properties of polymer liners by using advanced material formulations, surface modifications, nanocomposite reinforcements, and innovative fabrication techniques.

Modern hydrogen tanks consist of multiple layers and feature a polymeric inner layer that directly contacts hydrogen and must be capable of withstanding a wide range of temperatures from −40 to 80 °C and hydrogen pressures between 60 and 800 bar [8]. In this scenario, classical molecular dynamics (MD) simulations serve as a valuable complement to experimental methods, effectively modeling the static and dynamic behaviors of systems at the atomic level across various operating conditions [9,10,11,12,13]. Based on Newtonian mechanics, MD simulations allow for the prediction of atomic movement over time based on the principles of interatomic interactions [14]. These computer-based experiments share similarities with traditional laboratory experiments, involving processes such as sample preparation, statistical analysis, and validation, while allowing for adjustments to parameters such as temperature, pressure, and concentration [15]. This capability is particularly beneficial when testing extreme conditions [16] because MD simulations are more cost-effective and safer than laboratory experiments. Consequently, modeling can yield crucial insights into the molecular mechanisms and guide experimentalists towards the most promising systems for further exploration.

This review provides an in-depth overview of the latest advancements in the fabrication, properties, and hydrogen gas barrier performance of polymer liners and their composites, specifically for type IV hydrogen storage tanks. It emphasizes the significance of molecular modeling in predicting the material behavior and supporting the creation of next-generation high-barrier polymeric materials. Additionally, this review identifies key challenges and outlines future research directions to promote further enhancements in hydrogen storage technology.

## 2. Fabrication Processes and Properties

### 2.1. Common Polymers Used (HDPE, PA6, PA11, EVOH)

Common matrix materials utilized for type IV hydrogen tank liners include polyamide 6 (PA6), high-density polyethylene (HDPE), and several other polyether derivatives [17]. However, these common matrices require modifications to endow them with essential characteristics such as strength, aging resistance, barrier properties, and thermal stability to address complex failure factors and fully leverage their lightweight advantages [18]. Methods such as adding functional fillers, designing specialized microstructures, and refining processing techniques can be employed to optimize the properties of these materials [19]. Among the most effective strategies for enhancing performance is the incorporation of targeted modifiers, including large-surface-area flake fillers [20], high-performance polymers [21], fibers [22], nanomaterials [23], and anti-aging agents [24]. Adjusting the phase interface after adding heterogeneous materials to prevent adverse effects is crucial. Additionally, optimizing the synthetic conditions, such as the temperature, velocity, and pressure, is important to improve the aggregated state structure [25], which includes aspects such as crystallinity, free volume, and intergranular defects. In response to these evolving requirements, new processes are being developed to create suitable microstructures that achieve these goals.

**Figure 1:**
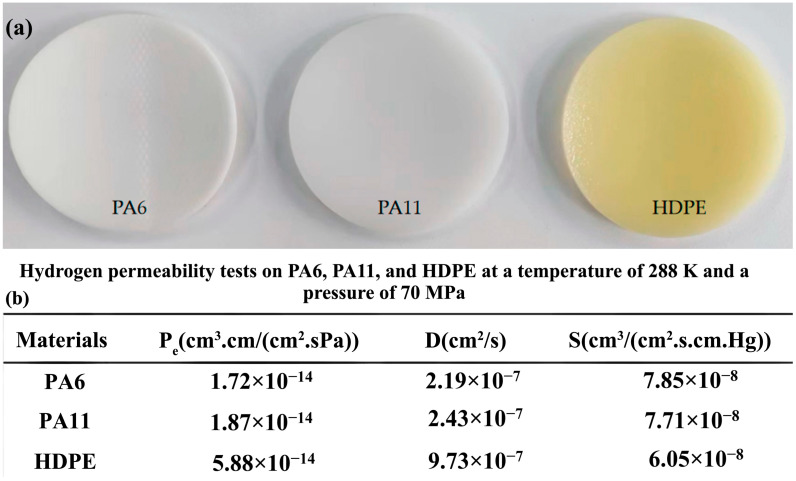
(**a**) Hydrogen permeation test samples and (**b**) hydrogen permeation test results of different materials [26]. Copyright 2023 MDPI.

Dong et al. [26] conducted a comprehensive study to evaluate the hydrogen gas permeability of PA6 as a liner material for type IV onboard hydrogen storage cylinders. Samples consisting of PA6, PA11, and HDPE were sourced from a manufacturer and prepared in accordance with the GB/T 42610-2023 standards [26]. Round specimens were precision-cut from the injection-molded liner weld to achieve a consistent diameter of 78 ± 1 mm. To ensure optimal testing conditions, the surfaces of the samples were meticulously cleaned with sandpaper, as illustrated in Figure 1a. A specialized hydrogen gas permeation test device was utilized to conduct experiments under controlled conditions, with pressures ranging from 0.1 to 99 MPa and temperatures between 233 and 373 K. The testing protocol included careful installation, sealing, temperature adjustment, and pressure monitoring. The permeability of PA6 differed significantly from those of PA11 and HDPE. PA6 exhibits slightly better hydrogen gas permeation resistance than PA11, which is reflected in its lower hydrogen gas permeability coefficient. Specifically, PA11 showed approximately 8.7% higher permeability than PA6. Conversely, HDPE demonstrated the lowest resistance among the three materials, with a permeability coefficient of approximately 242% that of PA6. Moreover, the diffusion coefficients of PA6, PA11, and HDPE followed similar patterns, with PA11 and HDPE exhibiting approximately 12.5% and 350% higher diffusion rates, respectively, relative to PA6 (Figure 1b).

Sun et al. [5] investigated the potential of PA6 filled with lamellar inorganic components (LIC) as a liner material for type IV hydrogen storage tanks to address the challenges posed by PA6’s high hydrogen gas permeability. A LIC/PA6 composite was prepared by combining a silane coupling agent, ball milling, and twin-screw extrusion. LIC/PA6 exhibited enhanced thermal and mechanical properties compared with regular PA6. Specifically, LIC/PA6 has a lower melting point of 218.0 °C, indicating altered crystallinity. The tensile strength, bending strength, and bending modulus of LIC/PA6 improved by 36%, 17%, and 12% relative to that of PA6, respectively, while maintaining a high elongation at break of over 200%, which ensured good toughness. LIC/PA6 exhibited a significantly reduced hydrogen gas permeability coefficient, achieving values as low as 4.7 × 10^−17^ mol/m·s·Pa at −10 °C and 50 MPa, and a maximum of 6.1 × 10^−16^ mol/m·s·Pa at 85 °C and 25 MPa, representing an improvement in barrier properties of approximately three to five times compared to those of PA6 under equivalent conditions.

**Figure 2:**
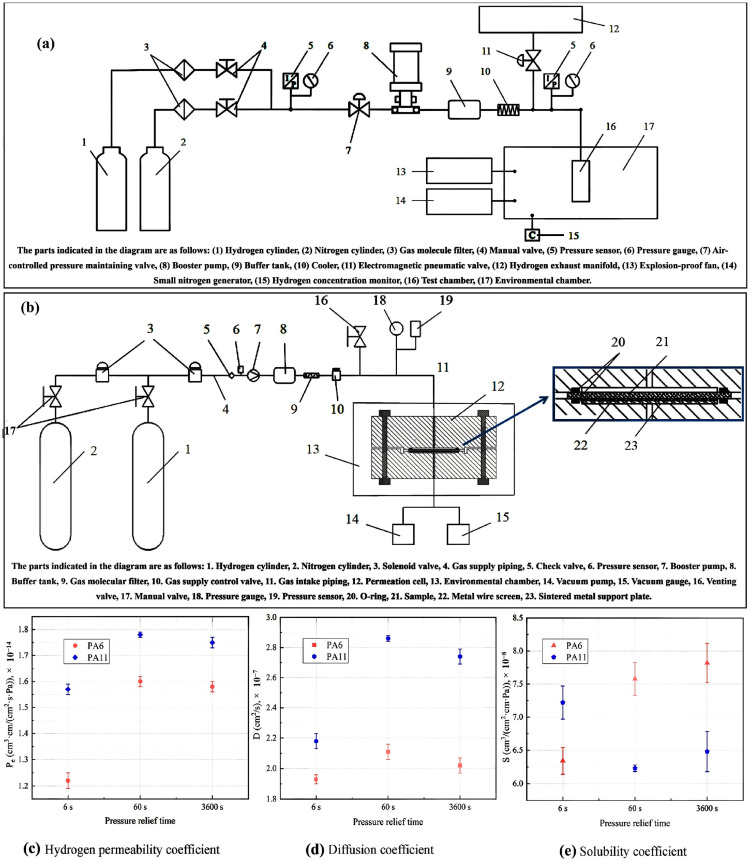
(**a**) Schematic illustration of the hydrogen cycle testing apparatus, (**b**) schematic illustration of the hydrogen permeation testing apparatus, and (**c**–**e**) results of hydrogen permeation tests for each sample post hydrogen cycle testing at various pressure relief times [27]. Copyright 2024 Elsevier.

Li et al. [27] studied the influence of pressure relief duration on the performance of liner materials subjected to various pressure cycling conditions. Figure 2 provides a detailed overview of the test sample preparation, sample pre-treatment, testing apparatus, and methodology used in their study. The PA6 and PA11 liners were sourced from a reputable manufacturer, minimizing the variability in composition and molding conditions. Rectangular samples of PA6 were cut using an angle grinder and shaped into round samples for hydrogen gas permeation tests and bone-rod samples for tensile tests using AutoCAD and a numerical control display. After cooling with a high-pressure water gun, the samples were polished using 500-mesh sandpaper, followed by 1000-mesh sandpaper to smooth the rough edges. These steps ensured compliance with the GB/T 42,610-2023 standard for hydrogen gas permeation tests regarding the contact area and thickness [27]. Tensile test samples measuring 170 mm in length and 20 mm in width were prepared according to the GB/T 1040.2–2022 specifications [27]. This meticulous preparation ensured the physical and chemical stability of the test materials. The PA6 and PA11 monomers exhibit strong hygroscopic properties because of their polar amine and carbonyl groups. To minimize the impact of humidity on the hydrogen gas permeation tests, the samples were placed in a vacuum drying oven at 1000–5000 Pa and 338 K until they were fully dried. Their weights were measured every 24 h, and a change of less than 0.1% of the initial weight indicated a fully dry state.

The material hydrogen cycle test device utilized in this study, independently developed by the China Special Equipment Inspection and Research Institute, was designed to conduct hydrogen cycle tests on polymer materials within a pressure range from 0.1 to 87.5 MPa and a temperature range from 233 to 373 K. Figure 2a illustrates a schematic diagram of the device. The testing procedure began with the preparation of round and bone-rod samples according to specified requirements, which were then placed in a temperature-controlled chamber. The environmental chamber maintains a control accuracy of ±1 K, ensuring precise testing conditions. After applying vacuum-sealing silicone grease to the sealing ring of the chamber, the setup was purged with nitrogen to eliminate residual gases before being switched to hydrogen for testing. The system allows for automated control of pressure cycling with predetermined upper and lower pressure limits, and the pressure relief time was adjusted based on the test conditions. Continuous pressure monitoring throughout the test cycles ensured that the samples were subjected to the intended conditions, thereby facilitating the evaluation of their performances in simulated real-world applications involving hydrogen exposure and pressure fluctuations.

Figure 2b presents a detailed schematic of the hydrogen gas permeation device, which is integral to conducting hydrogen gas permeability tests on materials such as PA6 and PA11. The device comprises several key components, including a permeation cell, which is divided into high-pressure and low-pressure chambers where the test samples are positioned. Hydrogen was introduced into the high-pressure chamber via a high-pressure filling system that included a hydrogen cylinder, booster pump, and buffer tank. The low-pressure chamber was equipped with a vacuum gauge to monitor the pressure changes crucial for assessing hydrogen diffusion through the samples. The system incorporates a pressure-relief mechanism with a venting valve to maintain safety during high-pressure operations. Additionally, environmental control was ensured through a constant temperature and humidity chamber, whereby precise conditions were maintained to enable accurate permeability measurements. This setup allows for real-time observation and calculation of hydrogen gas permeation parameters while ensuring the integrity of the results by minimizing fluctuations in the experimental conditions, making it essential for evaluating the performance of polymer materials in hydrogen environments.

The results presented in Figure 2c–e indicate that the hydrogen gas permeability coefficients of the PA6 and PA11 samples demonstrated significant variability based on the pressure relief times utilized during the hydrogen cycle tests. Specifically, after 50 cycles, both materials showed stable permeability coefficients within a 2% range when subjected to relief times of 60 and 3600 s. Conversely, a drastic drop in permeability of over 20% was observed for both materials when the relief time was reduced to 6 s, highlighting the detrimental effect of rapid decompression on hydrogen gas permeation. The permeability coefficient of PA6 was measured to be 1.60 × 10^−14^ cm^3^/(cm^2^·s·Pa) for 60 s and 1.22 × 10^−14^ cm^3^/(cm^2^·s·Pa) for 6 s. PA11 exhibited similar results, underscoring that quick pressure release significantly disrupts the internal structure and hydrogen transport properties of the materials.

Lee et al. [1] explored an innovative material blend aimed at enhancing hydrogen tank liners, emphasizing weight reduction and improved gas barrier properties. This blend incorporated PA6 and EVOH (PA6/EVOH) to achieve superior hydrogen gas resistance. Additionally, EPDM rubber was used to enhance the moldability of the composite material. To create a homogeneous ternary polymer blend, PA6, EVOH, and EPDM pellets were dry-mixed and processed using an extrusion machine at temperatures exceeding their melting points. The extrusion conditions, including the temperature and screw speed, were carefully optimized. After extrusion, the resulting materials were chopped using a pelletizing machine and injected into molds using a mini-injection molding machine. The injection molding process involved setting the injection temperature to 260 °C after a 10-min heating period in the microcompounder, followed by a 90 s injection time (inclusive of a 30 s dwell time) and a mold temperature of 60 °C. The injection pressure was regulated using an air valve with a setting of 1.3. Finally, the fabricated materials were molded after cooling to room temperature. Differential Scanning Calorimetry (DSC) was used to investigate the thermal behavior, rheometry was used to examine the processability, and FE-SEM was used to observe the morphological structures. The mechanical properties were measured using a universal testing machine (UTM), and the hydrogen gas permeability was tested using a gas transmission rate tester. The main findings from the study of the ternary polymer blends of PA6, EVOH, and EPDM are as follows.

**Figure 3:**
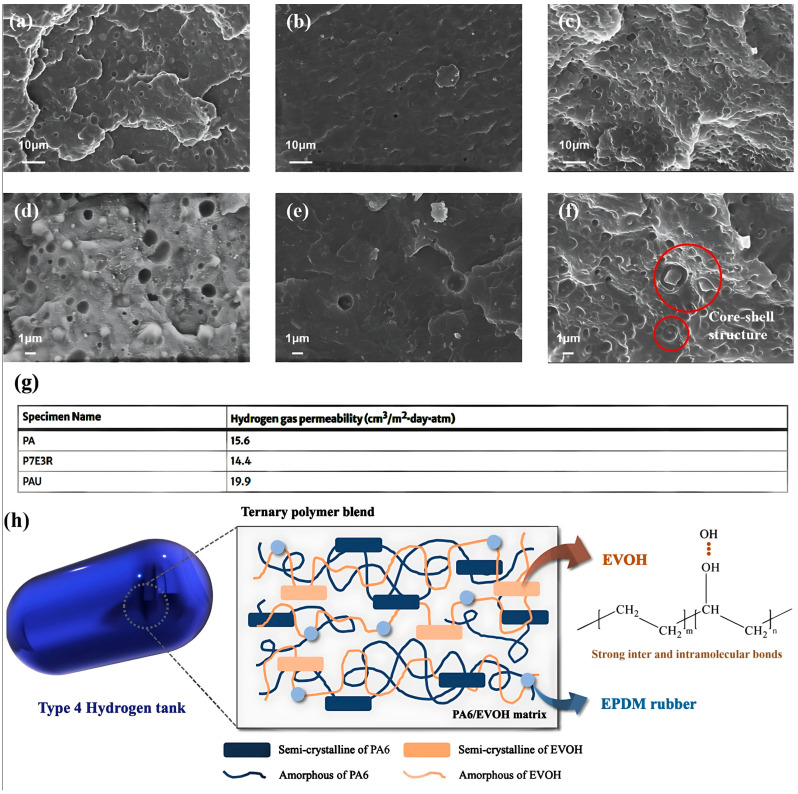
Morphological examination of PA6-based composites: (**a**,**d**) neat PA6; (**b**,**e**) P7E3; (**c**,**f**) P7E3R; (**g**) hydrogen gas barrier properties of ternary polymer blends; and (**h**) illustration of the mechanism behind improved gas barrier properties in PA6/EVOH/EPDM ternary blends [1]. Copyright 2024 Springer.

- Mechanical properties: The addition of EVOH significantly enhanced the tensile strength of the blends. Particularly, adding 10 wt% EVOH led to a 159% increase in the tensile strength for the optimized composition (70 wt% PA6 and 30 wt% EVOH, referred to as P7E3). However, the elongation at break decreased with increasing EVOH content, particularly beyond 50 wt%, owing to the semicrystalline nature of EVOH. Additionally, incorporating EPDM rubber into the optimized P7E3 blend improved the elongation at break, thereby enhancing processability, which is essential for applications such as hydrogen tank liners.
- Morphological characteristics: Morphological characterization was conducted to examine the miscibility and structural characteristics of the ternary polymer blends, as depicted in Figure 3. To obtain precise insights into the morphology of each polymer within the blends, etching was performed for 4 h using dimethylformamide for the P7E3 blend and xylene for the P7E3R blend. As shown in Figure 3a,d neat PA6 exhibited a distorted surface with noticeable pores resulting from the intrinsic moisture in the material. Conversely, the PA6/EVOH blend exhibited a significant reduction in porosity, suggesting enhanced compatibility between the two polymers (Figure 3b,e). Additionally, the ternary blends revealed a distinct core–shell structure owing to the differing hydrophilic and hydrophobic properties of the components, as illustrated in Figure 3c,f. The hydrophilic EVOH pellets effectively coated the EPDM rubber particles, contributing to the improved miscibility and elongation at break. This configuration minimized the interfacial area between the matrix and rubber, which possess inherently different characteristics, thereby enhancing the mechanical properties of the composite.
- Thermal behavior: DSC analysis indicated that the ternary blends exhibited single melting (T_m_) and crystallization (T_c_) temperatures, reflecting good miscibility among the polymers. The presence of EPDM affected the crystallization process, as evidenced by the reduction in the melting and crystallization enthalpies with its addition.
- Rheological properties: Rheological tests indicated that the addition of EPDM rubber particles slightly increased the viscosity of the blends, enhancing their solid-like characteristics necessary for maintaining the structure during injection molding.
- Gas barrier performance: The developed ternary polymer blends exhibited superior mechanical and hydrogen gas barrier properties compared to a commercial reference material, indicating their suitability for use as hydrogen tank liners (Figure 3g). This improvement indicates the potential for material thickness and weight reductions in liner applications.
- Mechanism illustration: The enhanced gas barrier properties of the PA6/EVOH/EPDM ternary blends are attributed to several key structural features (Figure 3h). The semi-crystalline arrangement of the PA6/EVOH matrix contributed to a longer tortuous path for gas diffusion, effectively reducing its permeability. Moreover, the strong inter- and intramolecular interactions formed by the hydroxyl groups in EVOH enhanced the gas barrier performance of the material by restricting polymer chain movement. Additionally, the incorporation of EPDM rubber, which is characterized by its cold resistance, benefits the overall integrity and impact strength of the blend. EVOH also played a critical role in coating the EPDM particles, improving its miscibility and overall performance, and making the ternary blend system a promising candidate for hydrogen tank liner applications owing to its superior mechanical and gas barrier characteristics.

### 2.2. Polymer Composites and Nanocomposites

To enhance the hydrogen barrier properties of polymer liners used in Type IV storage tanks, there has been a significant focus on the development of polymer composites and nanocomposites. Researchers are incorporating high-aspect-ratio nanofillers, such as graphene, MXenes, and layered silicate clays, into polymer matrices to create intricate pathways that impede hydrogen diffusion. These fillers not only improve the essential barrier properties but also enhance thermal, mechanical, and aging resistance attributes critical for the demanding conditions associated with hydrogen storage. The effectiveness of these materials is greatly influenced by factors such as filler dispersion, orientation, interfacial bonding quality, and overall filler content. This section explores the latest advancements in nanocomposite fabrication and evaluates the contributions of various nanofillers to reducing hydrogen permeability and improving the structural integrity of polymer liners.

Li et al. [28] explored the enhancement of PA6 properties through the incorporation of graphene (Gr) and a nano-two-dimensional lamellar filler, aiming to optimize its performance as a liner material for type IV hydrogen storage tanks. Initially, PA6 was dried in an oven at 80 °C for 8 h. Various mass fractions of graphene were carefully weighed. The PA6/Gr composites were created using melt blending via a twin-screw extruder, with the temperature settings for each zone being 230, 240, 245, 245, 240, and 240 °C while maintaining a rotational speed of 300 r/min. After extrusion, the resulting material was harvested, cooled with water, and processed using a pelletizer. The pellets underwent another drying phase in the oven at 80 °C for 8 h. Finally, these pellets were injection-molded to produce standard mechanical test samples and flakes for helium permeation testing, with the injection temperatures set at 245 °C for the first two zones, 235 °C for the next two zones, and finally 230 °C. Throughout this process, the injection and holding pressures were maintained at 58 MPa. This meticulous preparation process aimed to achieve a uniform dispersion of graphene within PA6, which is critical for enhancing the mechanical, crystalline, and gas barrier properties of the nanocomposites, ultimately contributing to their suitability for hydrogen storage applications.

**Figure 4:**
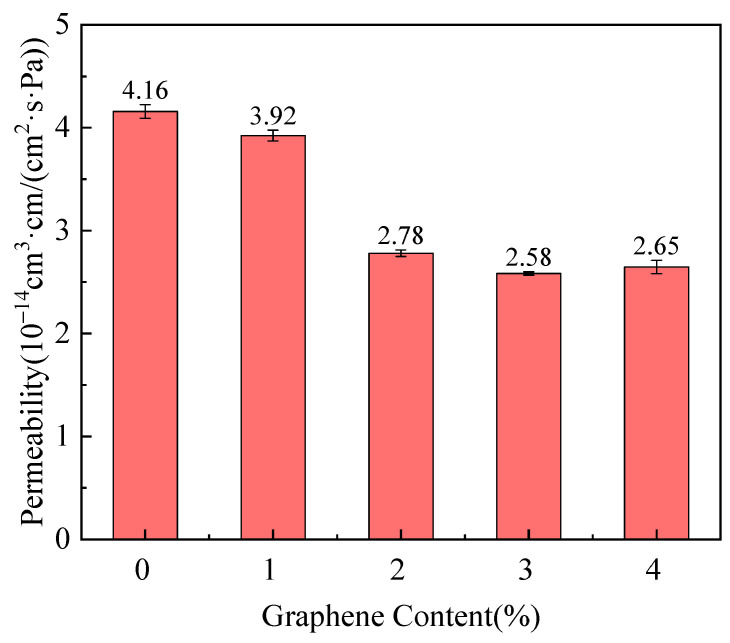
Influence of graphene content on the helium permeability coefficient of PA6/Gr. nanocomposites [28]. Copyright 2025 MDPI.

The composites with 2.0 wt% graphene exhibited the best overall performance, with improved crystallinity, impact strength, and helium permeability. Figure 4 shows the relationship between the helium permeability coefficient and the graphene content in PA6/Gr nanocomposites. As the graphene content increases, the helium permeability coefficient decreases, with the most significant reduction occurring between 1.0 and 2.0 wt% graphene. The permeability coefficient approaches 2.78 × 10^−14^ cm^3^/(cm^2^·s·Pa) at 2.0 wt% graphene, reflecting a 33.2% reduction compared to pure PA6. This improvement in gas barrier performance was attributed to the enhanced crystallinity of the composites and the increased thickness and lamellar structure of the graphene layers, which collectively contributed to establishing a more effective barrier against gas diffusion. However, when the graphene content exceeds 2.0 wt%, any further reduction in permeability became negligible because of the observed graphene agglomeration and stacking, which limited its effectiveness as a barrier.

Kis et al. [29] investigated the viability of PA6/organoclay-modified montmorillonite (OMMT) nanocomposites (NCs) as liner materials for type IV composite-overwrapped hydrogen storage pressure vessels. They studied the mechanical properties of PA6/OMMT NCs with different filler concentrations over a temperature range pertinent to hydrogen storage conditions (from −40 to 85 °C). Pristine PA6 powder was selected for its suitability for hydrogen composite overwrapped pressure vessel production, and montmorillonite organoclay was used as the filler at various concentrations of 1, 2.5, 5, and 10 wt%, considering that the surfactant content of montmorillonite is approximately 30–32 wt%. The preparation involved an extrusion injection process in which appropriate amounts of OMMT were effectively mixed with PA6. This mixture was melt-compounded using a twin-screw extruder to ensure a homogeneous distribution of the clay within the polymer matrix. After extrusion, the resultant filaments were granulated to a uniform size, and pristine PA6 and PA6/OMMT NCs were dried at 80 °C for 6 h to reduce moisture content. Subsequently, dog bone-shaped specimens were produced via injection molding, adhering to ISO 527 standard dimensions to ensure consistency for the subsequent mechanical testing [29].

**Figure 5:**
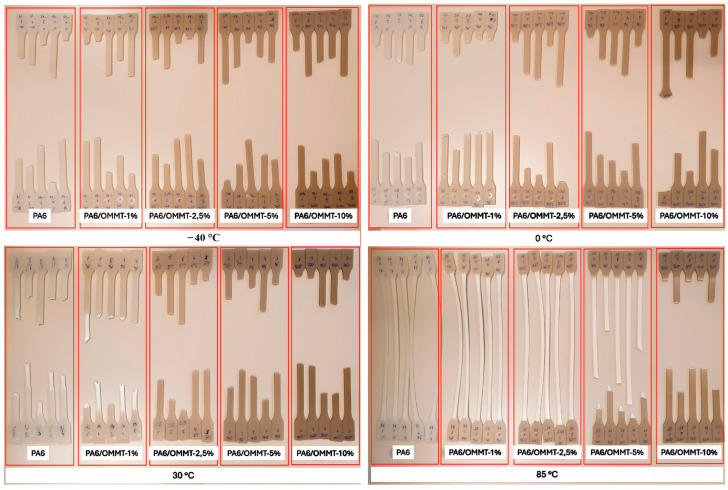
Visual representation of tensile specimens following testing conducted at temperatures ranging from −40 °C to 85 °C [29]. Copyright 2024 MDPI.

This study addressed the fundamental question of how the temperature and composition of OMMT influence the yield deformation of PA6 within the temperature range relevant to hydrogen storage tanks. As depicted in Figure 5, visual inspection of the tested specimens revealed the interplay between temperature and clay content. Notably, lower temperatures (between −40 and 0 °C) result in more brittle fractures for all specimens. Conversely, as the temperature was increased to between 30 and 85 °C, ductility typically increased; however, this trend was not consistent for specimens with higher clay contents at specific temperatures. Notably, specimens with 10 wt% OMMT exhibited brittle behavior at all temperatures, whereas those with a lower OMMT content (1 wt%) displayed a ductility comparable to that of neat PA6. The variations in the fracture appearance among the five specimens under each testing condition can be linked to the intrinsic material heterogeneity and small differences in processing and alignment. These factors may lead to minor inconsistencies in the fracture behavior, despite maintaining the same testing conditions. Increasing the OMMT content enhanced the Young’s modulus and decreased the impact strength; a 1 wt% OMMT content notably improved the yield strength at all the temperatures tested.

These findings demonstrate that the sensitivity of the Young’s modulus and yield stress to temperature is non-linear, with dynamic mechanical analysis (DMA) measurements confirming better yielding behavior and stiffness for the 1 wt% OMMT NC. However, an excessive clay content may result in brittleness, jeopardizing the safety and durability of storage tanks. Therefore, 1 wt% OMMT is the optimal formulation that balances the enhanced mechanical properties without excessive brittleness, thereby supporting the structural integrity of hydrogen storage systems. This study calls for further investigations, including thermal and permeability analyses, to fully characterize and optimize these materials for practical applications.

## 3. Molecular Insights into Hydrogen Transport and Permeability

Molecular dynamics (MD) simulations are essential for understanding and predicting the hydrogen barrier properties of polymer materials at the molecular level [30]. These simulations facilitate the detailed modeling of gas–polymer interactions, providing insights into the diffusion, dissolution, and permeation of hydrogen molecules within various polymer matrices. By simulating atomic-scale movements and intermolecular forces, MD methods accurately calculate key transport parameters, including the diffusion coefficient (D), solubility coefficient (S), and permeability coefficient (P_e_). This capability enables researchers to virtually screen materials, optimize molecular architectures, and identify effective polymer or composite systems prior to experimental testing, thus expediting the development of hydrogen storage materials.

Recent studies utilizing MD simulations have shed light on hydrogen transport dynamics in common polymers such as PA6 and HDPE. For instance, findings indicate that PA6 has lower diffusion and permeability coefficients than HDPE due to its denser molecular structure and stronger hydrogen bonding, which enhances its barrier performance. Conversely, HDPE’s semi-crystalline structure and non-polar backbone contribute to greater hydrogen diffusivity, as supported by both simulation and experimental results.

Additionally, MD simulations have proven effective in assessing the influence of nanofillers, like graphene, on the barrier properties of polymers. Simulations involving graphene-reinforced PA6 composites reveal that incorporating graphene sheets increases the tortuosity of the hydrogen diffusion pathways, resulting in a significant decrease in D and P values. These simulations also visualize filler-matrix interactions and help determine the optimal filler loading to maximize barrier efficiency without sacrificing mechanical flexibility.

Overall, these MD studies not only confirm experimental results but also facilitate the investigation of structure–property relationships, serving as a powerful tool for the rational design of next-generation hydrogen barrier materials [9].

### 3.1. Analysis of Factors Affecting Barrier Properties Using MD Simulations

#### 3.1.1. Temperature and Pressure

The growing need for high-pressure hydrogen storage in fuel cell vehicles and other energy applications necessitates the development of advanced polymer liners and thermoplastic composites with superior hydrogen gas barrier properties. Because of its small size and high diffusivity, hydrogen gas poses significant containment challenges in polymeric materials, which makes understanding its molecular interactions with polymer chains crucial for effective material design. Computational modeling techniques, particularly MD simulations, can provide insights into hydrogen transport mechanisms, polymer chain mobility, and gas permeability behavior. Theoretical models also aid in optimizing material composition for better hydrogen gas resistance. This section explores the molecular aspects of hydrogen gas permeability in polymeric systems, focusing on computational methods and their roles in enhancing the performance of hydrogen storage materials.

**Figure 6:**
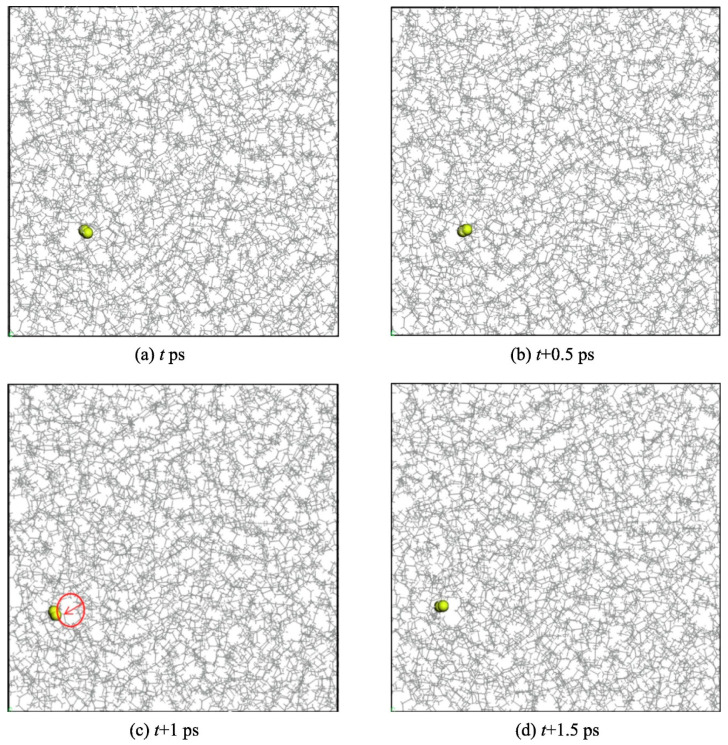
Diffusion mechanism of the hydrogen molecule in amorphous PE: (**a**) Initial position of the H_2_ molecule, (**b**) H_2_ molecule vibrating within a pore, (**c**) H_2_ molecule hopping to an adjacent pore, and (**d**) H_2_ molecule residing in a new pore after hopping [30]. Copyright 2022 Elsevier.

Zheng et al. [30] studied the permeation and diffusion of hydrogen in polyethylene (PE) to mitigate issues such as hydrogen embrittlement, which is commonly associated with metal pipelines. Their research employed GCMC and MD simulations to examine the hydrogen gas permeation in PE pipelines. The analysis focused on the solubility (S) and diffusion (D) characteristics of hydrogen within amorphous PE over the temperature range of 270–310 K and pressure range of 0.1–0.7 MPa. A PE single chain with 500 polymerization degrees (C_1000_H_2002_) and H_2_ molecules were precisely constructed using an all-atom model. Their structures were optimized in 500 steps. Ten three-dimensional periodic amorphous PE cells, each containing five PE chains, were created based on Theodorou and Suter’s method [31], with the lowest energy cell being further optimized through 10,000 steps to eliminate unreasonable structures. Dynamic operations were employed for equilibrium, including a 500 ps NVT relaxation at 300 K, six annealing cycles from 300 to 600 K, and further NPT relaxation at varying conditions. The temperature and pressure were controlled using a Nose thermostat and a Berendsen barostat, employing a time step of 1 fs for MD simulations. The final amorphous PE cell measured 52.65 Å in each dimension and had a density of 0.795 g/cm^3^, which is close to expected values, indicating the model’s validity. The findings reveal that the solubility, diffusion, and permeability coefficients of H_2_ in amorphous PE are positively correlated with temperature, adhering to Arrhenius’ law; thus, these coefficients at different temperatures can be extrapolated using this relationship. Pressure exerts a minimal effect on the permeability coefficient, whereas temperature significantly influences it. Consequently, strategies such as burying urban gas PE pipelines deeper or implementing insulating layers to mitigate temperature fluctuations should be considered to reduce H_2_ permeability. Additionally, utilizing PE materials with higher crystallinity is advisable for enhancing hydrogen transport efficiency. The diffusion of H_2_ within PE follows a “hopping” mechanism, wherein H_2_ molecules vibrate within the free volume pores for extended durations, resulting in small displacements. In contrast, they make frequent, larger hops to adjacent pores, continuing this cycle of vibration and hopping, which ultimately facilitates their permeation through the PE matrix. Figure 6 illustrates the diffusion process of an H_2_ molecule within an amorphous PE cell, highlighting the distinct stages of diffusion. Initially, the H_2_ molecule is trapped in a pore where it vibrates, resulting in small displacements. As the temperature increases and the thermal motion of the PE chains facilitates the formation of channels between adjacent pores, the H_2_ molecule can perform rapid hops into adjacent pores, leading to larger displacements. This “hopping” mechanism characterizes the diffusion process, where the molecule vibrates within a pore before quickly moving to a neighboring pore, thus enabling significant overall movement through the amorphous structure over time. The free volume increased with increasing temperature, facilitating the diffusion of H_2_ molecules through PE.

Zhang et al. [10] investigated the diffusion properties of hydrogen in HDPE and EVOH at different pressures (2.5–10 MPa) and temperatures (from room temperature to 80 °C) using MD simulations. This study involves the development of molecular models and MD simulations to explore the dissolution and diffusion behaviors of H_2_ in HDPE and EVOH using Materials Studio. The models were created with the Visualize module, and geometric optimization was performed, achieving energy convergence to 6 × 10^−8^ kcal/mol to stabilize the molecular chains. The Amorphous Cell module was used to generate disordered unit cells with three-dimensional periodic boundaries, specifically measuring 17.8 Å for HDPE and 19.9 Å for EVOH. Each adsorption cell contained 12 stable molecular chains, while the diffusion cell included 12 H_2_ molecules alongside the polymer chains. EVOH’s numerous hydroxyl groups enhanced intermolecular forces and hydrogen bonding, resulting in a denser chain arrangement and better barrier properties.

The models underwent 25 annealing cycles from 27 °C to 527 °C (300–800 K), with geometric optimization after each cycle. The configuration with the lowest energy was subjected to NVT and then NPT for relaxation. The energy stabilized over time, indicating the models achieved a stable structure. The simulations used a 1.0 fs step size, with temperature and pressure regulated using the Anderson–Berendsen method, while the electronic potential was analyzed using the Ewald method. This study encountered several challenges in investigating the complex behavior of hydrogen permeability in polymers such as HDPE and EVOH. One of the primary difficulties was the need to accurately model the molecular interactions and dynamics of hydrogen molecules within the polymer matrices. This required the development of sophisticated simulation models that could capture the hierarchical structure of the polymers and their varying degrees of crystallinity. Additionally, achieving realistic simulation conditions that closely mimicked the high-pressure and elevated temperature environments of hydrogen pipeline operations posed significant constraints, as many existing studies had explored either lower pressure levels or different temperature ranges that were not aligned with actual operational settings. Despite these challenges, the study yielded significant results regarding the permeability of hydrogen in the selected polymers. The key findings and conclusions are as follows.

**Figure 7:**
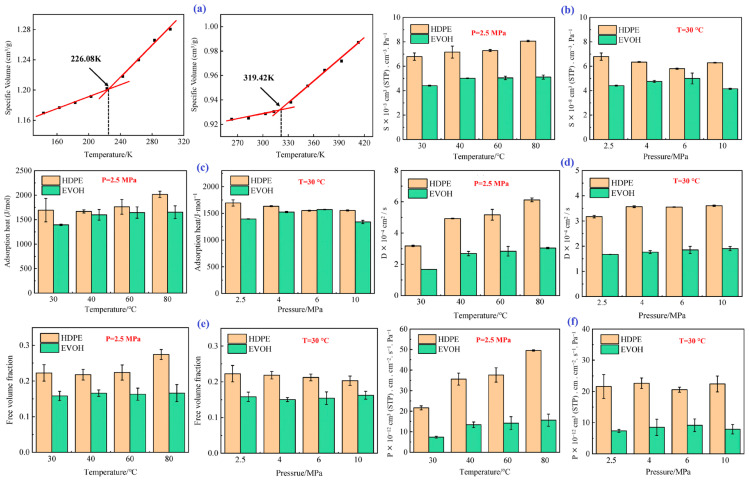
Characterization of hydrogen behavior in HDPE and EVOH at 2.5 MPa and 30 °C: (**a**) T_g_; (**b**) solubility coefficients; (**c**) isosteric heat of H_2_ adsorption; (**d**) diffusion coefficient; (**e**) FFV; and (**f**) permeability coefficient [10]. Copyright 2024 MDPI.

**Figure 8:**
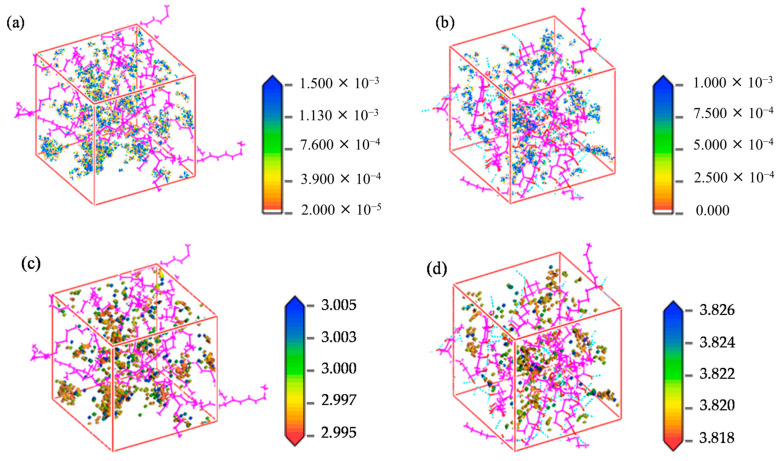
Distribution of hydrogen density field: (**a**) HDPE, (**b**) EVOH; isopycnic distribution: (**c**) HDPE; (**d**) EVOH [10]. Copyright 2024 MDPI.

- T_g_: As shown in Figure 7a, the T_g_ values of HDPE and EVOH are 226.08 and 319.42 K, respectively.
- Solubility coefficient: Figure 7b illustrates the solubility coefficients of hydrogen in HDPE and EVOH at 2.5 MPa and 30 °C. The hydrogen solubility in HDPE was significantly higher than that in EVOH. As the temperature increased from 30 to 80 °C, both materials exhibited increased solubility coefficients, with that of HDPE rising by 18.7% and EVOH by 15.9%, demonstrating a “reverse dissolution” behavior contrary to typical gas solubility trends. The solubility coefficients also showed different responses to pressure; that of HDPE initially decreased from 6.8 × 10^−8^ to 5.8 × 10^−8^ cm^3^ (STP)/(cm^3^·Pa), then slightly increased to 6.3 × 10^−8^ cm^3^ (STP)/(cm^3^·Pa). The solubility coefficient of EVOH increased from 4.4 × 10^−8^ cm^3^ (STP)/(cm^3^ Pa) to a peak of 5.0 × 10^−8^ cm^3^ (STP)/(cm^3^·Pa) at 6 MPa before decreasing to 4.1 × 10^−8^ cm^3^ (STP)/(cm^3^·Pa) at 10 MPa. As the temperature increased, the isosteric heat of adsorption increased, resulting in greater molecular motion and enhanced adsorption capacity (Figure 7c). Similarly, as the pressure increased, the isosteric heat in HDPE and EVOH aligned with the changes in the dissolution coefficient. Although the isosteric heat of H_2_ adsorption was nearly identical at 6 MPa, the solubility coefficients differed significantly because of factors such as entropy changes during solvation and interactions between the polymer and gas molecules. The nonpolar nature of hydrogen limited its interaction with the polymer chain, whereas the hydroxyl groups of EVOH formed hydrogen bonds that increased cohesion, thus restricting the polymer segment mobility and H_2_ permeation through the matrix.
- Diffusion coefficient: The calculated diffusion coefficients of H_2_ in HDPE and EVOH increased with increasing temperature and pressure, as illustrated in Figure 7d. When the temperature increased from 30 to 80 °C, the H_2_ diffusion coefficients in HDPE and EVOH increased by 92.9% and 81.6%, respectively. However, pressure had a negligible effect on the hydrogen diffusion coefficient, which remained consistent beyond a threshold of 4 MPa. The free volume fraction, indicating the unoccupied space within a polymer, increases with increasing temperature owing to polymer expansion, which enhances the diffusion coefficient for hydrogen (Figure 7e). Higher temperatures also improve the thermal motion of gas molecules, facilitating diffusion. Conversely, pressure changes had a negligible effect on the free volume fraction and diffusion coefficient. Notably, the diffusion coefficient of hydrogen in HDPE is approximately twice that in EVOH, primarily because the hydroxyl groups of EVOH lead to stronger hydrogen bonding and a reduced free volume, which hinders diffusion.
- Permeability coefficient: The permeability coefficient of H_2_ was higher in HDPE than in EVOH, reflecting similar trends in the diffusion and solubility coefficients (Figure 7f). Both materials showed significant increases in permeability upon increasing the temperature from 30 to 80 °C; the permeability of HDPE and EVOH increased by 129.0% and 112.7%, respectively. Pressure had a negligible effect on permeability, with variations from 2.5 to 10 MPa resulting in changes of only 3.7% and 7.5% for HDPE and EVOH, respectively. In low-pressure environments, the gas permeability is primarily dependent on temperature rather than pressure or gas concentration.
- Permeability mechanism: The mechanisms by which H_2_ permeates through HDPE and EVOH share common features, including an aggregation adsorption process that occurs in the low-potential energy regions and a diffusion process characterized by transitions between holes driven by vibrational motion (Figure 8). During the adsorption phase, H_2_ molecules predominantly accumulate in low-potential energy areas, with EVOH displaying a particular affinity for regions devoid of hydrogen bonds. In the subsequent diffusion phase, H_2_ molecules vibrate within a specific hole before moving to another hole, where they continue to vibrate. Higher temperatures result in an enhanced range of motion and increased frequency of transitions for H_2_ molecules.

Fang et al. [12] used the Materials Studio software to study hydrogen gas permeation in the liner materials of type IV hydrogen storage vessels, specifically PE and PA6, at temperatures ranging from 263 to 353 K. They calculated the fractional free volume (FFV), solubility coefficient, diffusion coefficient, and permeability coefficient of hydrogen in materials. Their results showed that temperature significantly affected these properties: the solubility coefficient decreased with increasing temperature, whereas the diffusion and permeability coefficients increased. PE and PA6 are commonly used thermoplastic polymers with high molecular weights, but constructing large systems through molecular simulation is challenging. In this study, the PE unit cell has a molecular weight of 3630 and PA 3440, with polymerization degrees of 40 and 18, respectively. Higher chain articulation typically reduces hydrogen diffusion. The study also examines the effects of temperature and pressure typical of type IV hydrogen storage vessels on fractional free volume, solubility coefficient, and diffusion coefficient. Monomer models are created using the Visualizer interface and optimized with the COMPASS II force field. The “Amorphous Cell” module is then used to develop the amorphous unit cell, with initial densities of 0.9 g/cm^3^ for polyethylene and 1.1 g/cm^3^ for polyamide, employing periodic boundary conditions. The main conclusions drawn from this study are as follows.

**Figure 9:**
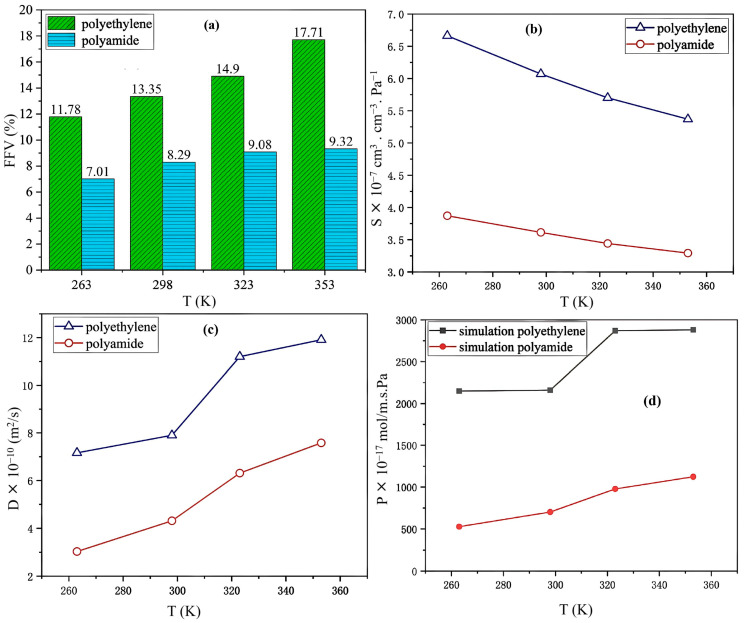
Histogram of FFV for PE and PA6 at various temperatures (**a**), the solubility coefficients (**b**) and the diffusion coefficients (**c**) of PE and PA6 at 30 MPa, and the permeability coefficients from (**d**) experimental data [12]. Copyright 2023 Elsevier.

- Free Volume: The histogram shown in Figure 9a illustrates the FFV of PE and PA6 at 263, 298, 323, and 353 K, indicating that the FFV for both materials increased with increasing temperature. Notably, at all temperatures, the FFV of PE consistently exceeded that of PA6. Specifically, the FFV of PE increased from 11.78% at 263 K to 17.71% at 353 K, reflecting an increase of 5.93%. In comparison, the FFV of PA6 increased from 7.07% to 9.322% over the same temperature range, which is a smaller increase of 2.315%. This demonstrated that the FFV of PE was significantly more sensitive to temperature changes than that of PA6.
- Solubility coefficient: The solubility coefficients of hydrogen in PE and PA6 decreased as the temperature increased. Within the temperature range of 263–353 K, PE consistently demonstrated a higher solubility coefficient than PA6 (Figure 9b).
- Diffusion coefficient: The diffusion coefficient showed a positive correlation with FFV, with both parameters increasing as the temperature increased (Figure 9c). As the temperature increased, the free volume of the system expanded, thereby increasing the effective diffusion space available for H_2_ molecules. This heightened thermal motion of the molecular chains in PE and PA6, along with the H_2_ molecules, enhanced the likelihood of diffusion, which, in turn, increased the diffusion coefficient.
- Permeability coefficient: Compared to PE, PA6 exhibited superior resistance to hydrogen and better gas permeability, with its ability to prevent hydrogen gas permeation improving by three to four times. Additionally, the permeability coefficient of PE was more significantly influenced by temperature changes than that of PA6 (Figure 9d).

Su et al. [32] explored the dissolution and diffusion of hydrogen in PA6 under the operational conditions of type IV hydrogen storage tanks, focusing on temperatures ranging from 233 to 358 K and pressures between 0 and 87.5 MPa. They employed MD and GCMC simulations to comprehensively analyze these phenomena. The key findings regarding the hydrogen gas permeability of PA6 as a liner material for type IV hydrogen storage tanks are as follows.

**Figure 10:**
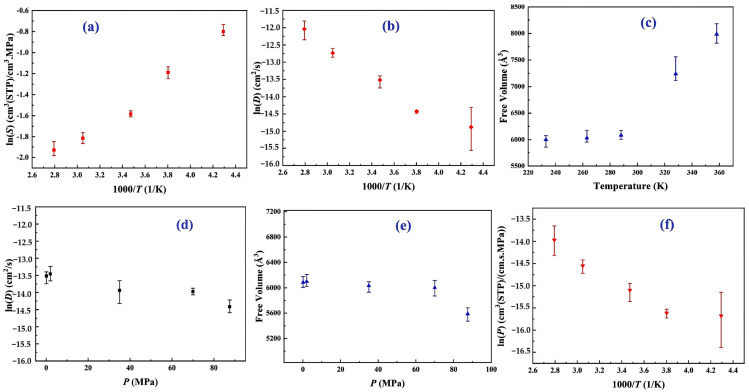
Comprehensive analysis of hydrogen behavior in PA6: (**a**) solubility coefficients of hydrogen in PA6 with 30% crystallinity at various temperatures; (**b**) diffusion coefficients of hydrogen in PA6 with 30% crystallinity at 0.1 MPa across a range of temperatures; (**c**) influence of temperature on the free volume within the cell; (**d**) diffusion coefficients of hydrogen in PA6 with 30% crystallinity at 288 K under different pressure conditions; (**e**) effect of pressure on the free volume in the cell; and (**f**) permeability coefficients of hydrogen in PA6 with 30% crystallinity at 0.1 MPa at various temperatures [32]. Copyright 2024 Elsevier.

- The solubility coefficients of H_2_ in PA6, with 30.00% crystallinity, exhibit Henry-type behavior that is distinct from gases with higher solubilities, such as CO_2_ [33]. Temperature negatively affects the solubility coefficient of H_2_ in PA6; as temperature increases, the thermal motion of the gas and polymer molecules rises, making gas adsorption more difficult. Consequently, this leads to a gradual increase in the impact of temperature on the solubility coefficient, resulting in a slower rate of change, as illustrated in Figure 10a.
- The results revealed a positive correlation between the temperature and the diffusion coefficient of H_2_ in PA6, which was attributed to the increased kinetic energy that facilitated diffusion within the polymer (Figure 10b). Multiple Arrhenius regions were observed in the diffusion coefficient relative to the temperature for the type IV hydrogen tanks. Additionally, the study assesses free volume in the polymer using a hard probe approach with a probe radius of 1.445 Å, representing the kinetic size of H_2_ (Figure 10c). Below the T_g_ of 323.01 K, the free volume remained nearly constant, whereas above the T_g_, it increased significantly, with the free volume at 358 K being 1.33 times higher than that at 233 K. This greater free volume enhanced gas diffusion, which was reflected in the elevated diffusion coefficient.
- The gas diffusion in PA6 is affected by pressure. Five characteristic pressures (0.1, 2.0, 35.0, 70.0, and 87.5 MPa) were analyzed at 288 K (Figure 10d). The findings indicated a slight decrease in the diffusion coefficient as the pressure increased, with the coefficient at 87.5 MPa being only 40.70% of that at 0.1 MPa. This suggests that, although pressure influences gas diffusion, the effect is limited, particularly within a narrow pressure range. Additionally, increased pressure compacted the polymer, resulting in a reduced free volume and more strenuous gas diffusion (Figure 10e), a phenomenon referred to as the hydrostatic effect. The permeability coefficient was slightly reduced at a constant temperature, correlating with the changes in the diffusion and solubility coefficients.
- This study presented the permeability coefficients derived from the solubility and diffusion coefficients at varying temperatures. Although the solubility and diffusion coefficients exhibit opposite trends with increasing temperature, the permeability coefficient increases overall (Figure 10f). This trend aligns with the findings from previous research, indicating that higher temperatures reduce the gas barrier properties of polymers [5]. The effect of temperature on diffusion was notably stronger than that on dissolution, resulting in a permeability coefficient that mirrored the diffusion coefficient trend. The presence of multiple Arrhenius regions suggests challenges in applying Arrhenius’ law to characterize hydrogen gas permeation in type IV tanks.

#### 3.1.2. Polymer Crystallinity and Molecular Chain Structure

When investigating the hydrogen permeability of PFSA membranes, Takeuchi et al. [34] experimentally noted that the polymer’s crystallinity restricts hydrogen transmission. Increased crystallinity diminishes the volume fraction of cavities within the polymer, thereby enhancing the gas barrier properties of the film. Similarly, Kane [24] showed, through calculations, that crystallinity in PE also affects hydrogen permeability, concluding that higher crystallinity results in a more effective gas barrier. Polyamide, being a semi-crystalline polymer, typically exhibits lower crystallinity compared to highly crystalline materials like polyethylene. Based on this differentiation, one would expect PE to possess superior gas barrier properties relative to PA. However, under identical experimental conditions, Klopffer et al. [35] found that the hydrogen permeability of PE100, with a crystallinity of 60%, was actually greater than that of PA11, which had a crystallinity of 20% (with permeabilities of 8.93 × 10^−16^ mol/(Pa·m·s) for PE100 and 3.57 × 10^−16^ mol/(Pa·m·s) for PA11 at 20 °C and 2 MPa). This counterintuitive result has also been reported by other researchers [36]. This finding suggests that factors influencing gas permeability in polymers extend beyond crystallinity and may also be linked to the cohesive energy density of the amorphous phase [35]. Polyamide contains polar structures capable of forming intramolecular and intermolecular hydrogen bonds, which enhance the interaction among molecular chains and consequently lower permeability. Due to the presence of these hydrogen bonds, Pepin et al. [37] reported that the hydrogen permeability coefficient of PA12 is five times greater than that of PA6, reaching 3.42 × 10^−15^ mol/(Pa·m·s) at 55 °C and 18 MPa. Additionally, the functional groups on the polymer’s side chains and the orientation of the molecular chains also play a significant role in influencing gas permeability; the former affects the mobility of molecular chains, while the latter is related to the formation process of the polymer. Smith examined the durability and hydrogen permeability of various polymers, including HDPE and PA, that were produced using different molding techniques during temperature cycling experiments [2].

**Figure 11:**
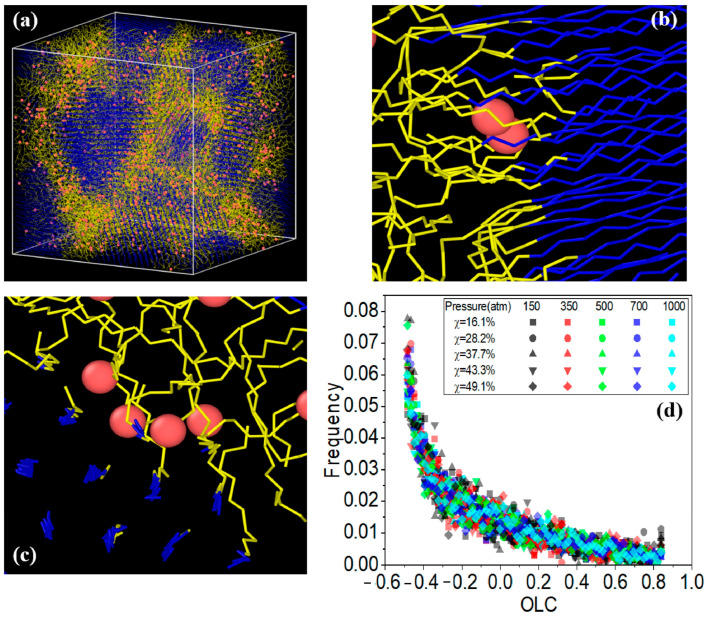
The final configuration of the GCMC/MD simulation for the HDPE system, with a crystallinity of χ = 49.3% and a pressure of 1000 atm, is presented in panel (**a**). The hydrogen molecules are depicted as dissolved within both the bonded crystalline–amorphous interface in panel (**b**) and the non-bonded crystalline–amorphous interface in panel (**c**). Additionally, the OLC histogram is displayed in panel (**d**) [38]. Copyright 2023 Elsevier.

Zhao et al. [38] analyzed the crystallization of PE with various chain structures using GCMC/MD simulations. They further investigated the solubility of hydrogen (H_2_) in both amorphous and semi-crystallized PE under typical operational pressures encountered in hydrogen storage devices, utilizing hybrid coarse-grained GCMC/MD simulations. To assess hydrogen solubility across different PE matrices, these hybrids were applied to amorphous, semi-crystalline, and partially crystallized HDPE systems at 300 K. In the simulations, GCMC exchanges were performed on coarse-grained hydrogen molecules (CGH_2_) to align the chemical potential with that of an imaginary hydrogen gas source at the specified pressure. Each MC step involved attempting to randomly insert or delete CGH_2_ molecules within the polymer matrix, with acceptance probabilities calculated based on energy changes using the Boltzmann equation. The MD segment modeled the movement of hydrogen and HDPE molecules over a total duration of 3 ns, amounting to 3 million MD steps, with 5000 MC steps completed for every 1000 MD steps. Five distinct pressures (150, 350, 500, 700, and 1000 atmospheres) were employed to reflect typical operational pressures in hydrogen containers while ensuring that the polymer maintained a constant temperature of 300 K throughout the simulations. The optimal crystallization temperature for the OPLS-UA coarse-grained PE was determined to be 380 K, resulting in approximately 50% crystallinity, which closely agrees with the experimental values. Branched PE systems exhibited limited crystalline phases owing to hindered crystallization. The total H_2_ solubility increased with pressure, with branch density significantly affecting solubility in the amorphous phase, whereas branch length had minimal impact. H_2_ molecules were primarily excluded from the crystalline phase, favoring the amorphous region and the crystalline–amorphous interface. As crystallinity increased, the H_2_ solubility decreased, and H_2_ tended to remain at the crystalline–amorphous interface, likely because of the constraints imposed by the crystalline phase and the presence of a rigid amorphous phase. Figure 11 depicts the results of the GCMC/MD simulation of the HDPE system with 49.3% crystallinity under a pressure of 1000 atm. Figure 11a shows the overall configuration, indicating a significant presence of hydrogen dissolved at the crystalline–amorphous interfaces. Specifically, Figure 11b reveals that hydrogen predominantly resides at the bonded crystalline–amorphous interface with an OLC between 0.8 and 0.9, suggesting strong spatial alignment with the crystalline structure. Moreover, Figure 11c illustrates the presence of hydrogen in the non-bonded crystalline–amorphous interface, indicated by an OLC of between −0.5 and −0.4, reflecting a less favorable interaction than the bonded interface. The histogram shown in Figure 11d quantitatively depicts the distribution of H_2_ molecules across these interfaces, highlighting their overall preference for dissolution in the bonded phase.

#### 3.1.3. Effect of Hydrogen Content

The amount of hydrogen present in the storage environment significantly affects the barrier performance of polymer liners and composites in Type IV hydrogen storage tanks. Increased hydrogen concentration, particularly at high pressures, can enhance solubility and diffusion within the polymer matrix, potentially compromising the material’s integrity over time and leading to greater hydrogen permeability. Moreover, elevated hydrogen levels may cause physical aging or swelling in certain polymers, further changing their transport properties. Therefore, grasping the relationship between hydrogen content and these crucial factors is vital for optimizing material selection and tank design. Ding et al. [13] examined the T_g_, diffusivity, and tensile properties of amorphous PE in high-pressure hydrogen environments through MD simulations. They assessed the influences of the temperature, hydrogen concentration, and pressure, all of which are vital for ensuring the safety of high-pressure hydrogen storage tanks. MD simulations were conducted using the open-source LAMMPS software [39]. After constructing and optimizing the model, energy minimization was performed with the CVFF force field. NPT dynamics were then run at 250 K and 0 atm pressure for 8 ns to equilibrate the system, ensuring stability in temperature, pressure, density, and energy [40]. Different amounts of hydrogen molecules were subsequently removed to create PE systems with varying hydrogen contents, keeping the structural integrity of the PE intact. PE systems with hydrogen quantities ranging from 0 to 175 molecules, in increments of 25, were established to examine the influence of hydrogen content, with temperatures set at 250, 300, and 350 K to reflect the operating range of high-pressure hydrogen storage tanks (−40 °C to 85 °C). The simulations also utilized six pressure settings (0, 150, 350, 500, 700, and 1000 atm) representative of typical operational pressures [38]. The key findings and conclusions of their study are as follows.

**Figure 12:**
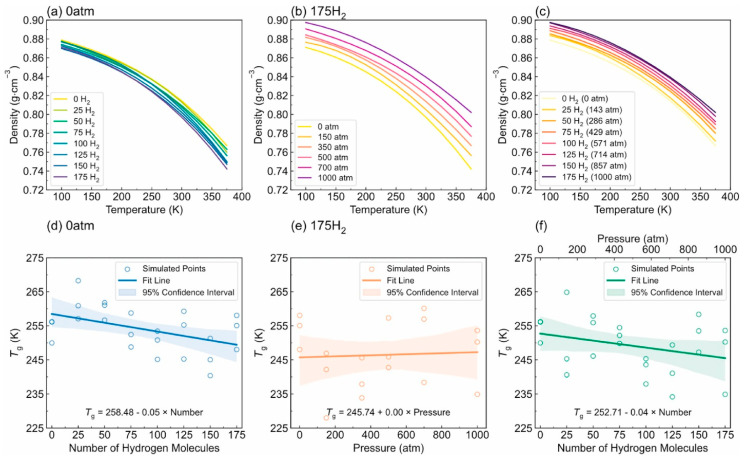
Density-temperature curves and T_g_ of polyethylene materials are analyzed under various conditions. The effect of hydrogen content on T_g_ (under constant pressure: 0 atm): (**a**) Density-temperature curves and (**d**) T_g_ of PE systems with various numbers of hydrogen molecules. The effect of pressure on T_g_ (under constant hydrogen content: 175 H_2_): (**b**) Density-temperature curves and (**e**) T_g_ of PE under various pressures. The coupled effect of hydrogen content and pressure on T_g_: (**c**) Density-temperature curves and (**f**) T_g_ of PE systems containing a corresponding quantity of hydrogen molecules under various pressures [13]. Copyright 2024 Elsevier.

**Figure 13:**
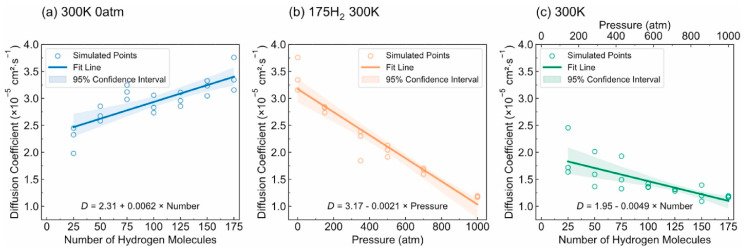
Diffusion coefficients of polyethylene in different conditions: (**a**) Effect of hydrogen content at 0 atm pressure. (**b**) Effect of pressure with 175 hydrogen molecules included. (**c**) Interaction effects of hydrogen content and pressure [13]. Copyright 2024 Elsevier.

**Figure 14:**
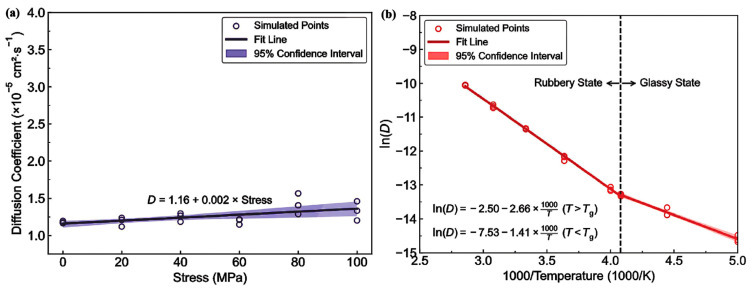
(**a**) Diffusion coefficients of H_2_ molecules in polyethylene under various tensile conditions. (**b**) Diffusion coefficients of H_2_ molecules in PE at different temperature levels [13]. Copyright 2024 Elsevier.

**Figure 15:**
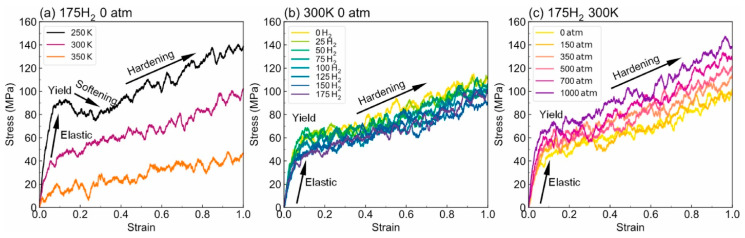
Stress–strain behavior of PE (**a**) under varying temperatures, (**b**) with different H_2_ contents, and (**c**) under different pressure conditions [13]. Copyright 2024 Elsevier.

**Figure 16:**
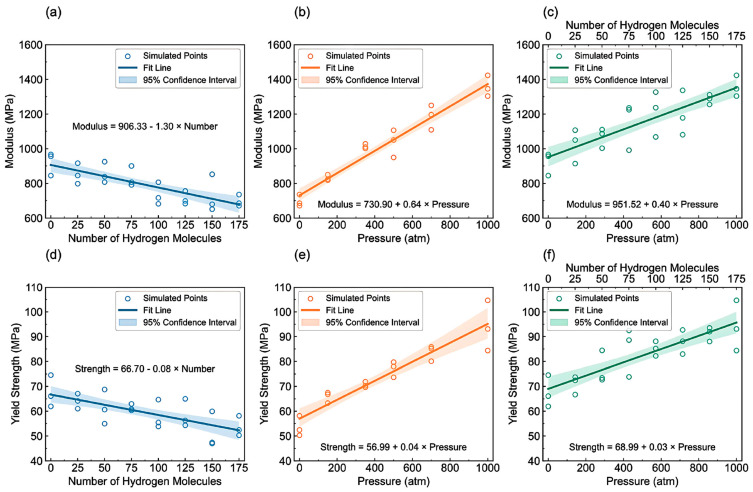
(**a**) Elastic modulus and (**d**) yield strength of polyethylene in relation to hydrogen molecule concentration at T = 300 K and P = 0 atm. (**b**) Elastic modulus and (**e**) yield strength of PE with 175 hydrogen molecules as a function of pressure at T = 300 K. (**c**) Elastic modulus and (**f**) yield strength of PE with varying amounts of hydrogen under different pressure conditions at T = 300 K [13]. Copyright 2024 Elsevier.

- T_g_: Figure 12 illustrates the complex interplay between the hydrogen content, pressure, and the resulting density–temperature characteristics and T_g_ of the PE systems. As depicted in Figure 12a, increasing the hydrogen content at a constant pressure (0 atm) results in decreased density, contributing to enhanced free volume and segmental mobility of the polymer. Figure 12d shows a corresponding significant reduction in T_g_, demonstrating a negative correlation attributed to the plasticizing effect of H_2_ molecules. Figure 12b reveals that under a constant hydrogen content (175 H_2_ molecules), increasing the pressure leads to an increase in density but does not significantly affect T_g_, as indicated in Figure 12e**,** where the slope is effectively zero. Furthermore, the coupled effects illustrated in Figure 12c,f show that although the hydrogen content and pressure influence the density and T_g_, the primary factor for T_g_ reduction remains hydrogen, with a slope of 0.04, highlighting that the plasticizing effect of hydrogen outweighs the compressive influence of pressure on T_g_, thus underscoring the critical role of hydrogen in modulating the thermal properties of PE in high-pressure environments.
- Diffusivity: The results illustrated in Figure 13 offer important insights into the diffusion characteristics of PE at different hydrogen contents and pressures. Figure 13a reveals a positive relationship between the hydrogen content and diffusion coefficient at 0 atm pressure, suggesting that an increase in the number of H_2_ molecules enhances the diffusion capacity of PE. This enhancement is attributed to the plasticizing effect, which increases the free volume and improves chain mobility. Conversely, Figure 13b indicates that under a fixed content of 175 H_2_ molecules, increasing the pressure results in a significant decrease in the diffusion coefficient, with a negative regression coefficient, demonstrating that the applied pressure restricts the free volume, thereby hindering the mobility of H_2_ molecules within the PE matrix. Furthermore, the coupled effects illustrated in Figure 13c confirm that when the hydrogen content and pressure is increased, the overall diffusion coefficient decreases, indicating that the negative impact of pressure outweighs the positive contribution of the increased hydrogen content, leading to reduced diffusion rates in practical applications. As shown in Figure 14a, under tensile stress conditions at constant temperature (300 K) and pressure (1000 atm), the diffusion coefficients of hydrogen in PE exhibit a nuanced response to tensile stress, suggesting enhanced molecular mobility. Figure 14b reveals that the diffusion coefficients vary with temperature, further emphasizing that higher temperatures facilitate the increased diffusion of H_2_ molecules within the PE, thus illustrating how tensile stress and thermal conditions significantly affect hydrogen mobility in high-pressure scenarios.
- Tensile properties: The findings from Figure 15 illustrate the complex interplay of temperature, hydrogen content, and pressure on the stress–strain behavior of PE. Figure 15a indicates that as the temperature increases from 250 to 350 K, the stress–strain curve transitions from a more pronounced elastic behavior to a bilinear response, indicating reduced stiffness and strength owing to weakened intermolecular interactions and enhanced chain mobility at elevated temperatures. Figure 15b shows the effects of increasing hydrogen content, where a higher number of H_2_ molecules correlates with decreased slopes in the elastic region, signifying a reduction in the elastic modulus and yield strength owing to the plasticizing effects of hydrogen that promote chain mobility and reduce intermolecular cohesion. Furthermore, Figure 15c emphasizes that increasing the pressure leads to significant enhancements in the elastic modulus and yield strength, as the tighter packing of the polymer chains and improved intermolecular interactions under high pressure counter the weakening effects of hydrogen. The results presented in Figure 16 further quantify these observations, revealing that the elastic modulus and yield strength of PE decrease linearly with the addition of H_2_ molecules while increasing markedly with pressure. This indicates a nuanced relationship in which the detrimental effects of hydrogen can be mitigated by the application of pressure, thereby improving the mechanical properties of PE in hydrogen-rich environments.

This study offers a thorough molecular-level insight into the individual and combined impacts of hydrogen and pressure on PE, which is essential for forecasting polymer behavior in high-pressure hydrogen scenarios. The capability to examine these effects independently through MD simulations provides valuable information that can inform the design of expensive experimental procedures. In contrast to earlier research, these findings elucidate the specific mechanisms by which hydrogen and pressure alter the mechanical properties of PE, thereby enhancing our comprehension of the primary factors that influence polymer performance in high-pressure hydrogen environments. Future investigations should aim to assess the effects of various polymer types and different stress conditions to evaluate the broader applicability of these results. Moreover, experimental validation of the MD simulation outcomes will be critical to verify the predicted behaviors and to further enhance our understanding of polymer performance under conditions of high-pressure hydrogen.

#### 3.1.4. Effect of High-Pressure Hydrogen Decompression

Rapid gas decompression (RGD) occurs when high-pressure gases like hydrogen are suddenly released, causing significant damage to polymers and composites through mechanisms such as cavitation, blistering, cracking, and delamination [41]. Factors influencing this damage include gas type, solubility, initial pressure, temperature, and the polymer’s mechanical properties. To mitigate damage, strategies involve using polymers with high T_g_, increased crystallinity, and reduced gas permeability, alongside multilayer structures for gas resistance. Limited literature exists on RGD, but earlier studies showed that oversaturated hydrogen can create internal bubbles, leading to cracks and enhanced gas permeation [42]. Initial studies by Yamabe et al. [43] explored the effects of decompression on various elastomers, while subsequent investigations by Jaravel et al. [44] and Kane-Diallo et al. [45] analyzed the impact of gas pressures and rates on damage progression. Thermoplastic materials, such as HDPE and PA [36], are also susceptible to RGD, with studies documenting cavitation and internal damage during high-pressure exposure [46]. The ratio of yield stress to Young’s modulus is critical in assessing pressure thresholds for liner collapse in these materials. Pepin et al. [47] conducted laboratory-scale experiments to investigate liner collapse during compression and decompression, using the thermoplastic polymer PA 6 (T_g_ 42 °C) in an autoclave at up to 30 MPa of hydrogen pressure and 50 °C. Their research identified key parameters essential for developing predictive models of this damage mechanism [37].

Zhao et al. [48] investigated the behavior of hydrogen in amorphous PE by specifically examining its diffusion across various PE matrices and the impact of rapid depressurization. Their comprehensive study utilized all-atom MD simulations to analyze the effects of hydrogen on PE, with particular emphasis on the tensile properties, Tg, and hydrogen diffusion characteristics. All simulations were performed using the large-scale Atomic/LAMMPS with the AIREBO-Morse potential, specifically designed for hydrocarbon systems, to ensure accurate results. Temperature and pressure were controlled using the Nose–Hover thermostat and barostat, focusing on polymer atoms to prevent overheating of hydrogen molecules due to their differing mobilities. The study utilized finite and infinite chain models of PE, with the standard chain length set to a degree of polymerization (DP_total_) of 2000, reflecting real-world polyethylene weights. Simulations included variations in chain length and structure, incorporating short, medium, and long branches to examine their effects on hydrogen diffusion. Additionally, plasticizers and 2D nanofillers, specifically a graphene layer, were investigated to understand their impacts on the properties of the PE matrix. The key findings and conclusions are as follows.

**Figure 17:**
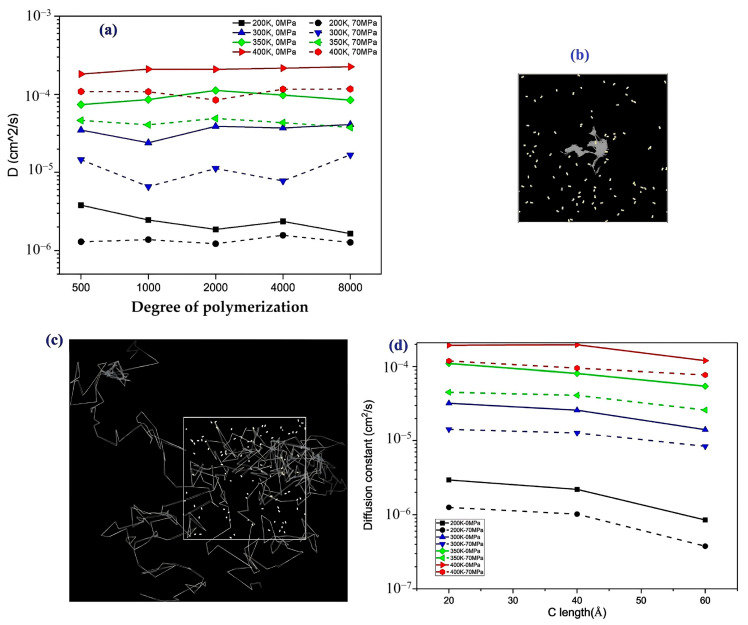
The relationship between the degree of polymerization (DP) and the diffusion constant (D) (**a**), the diffusion trajectory of hydrogen in DP2000 polyethylene at 200 K (**b**), the diffusion trajectory of H_2_ in the same material at 400 K (**c**), and the impact of chain orientation on the diffusion constant of hydrogen (**d**) [48]. Copyright 2022 Elsevier.

**Figure 18:**
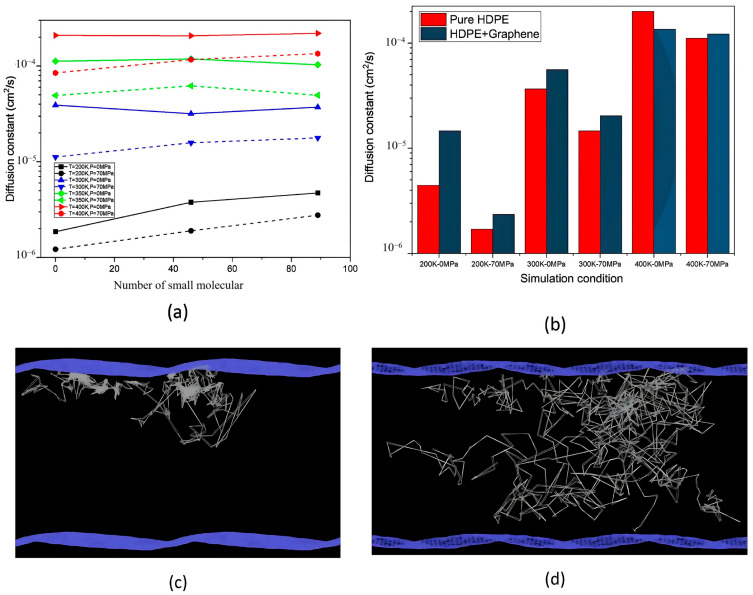
The influence of small molecules on H_2_ diffusion (**a**); the effect of a continuous graphene (Gr) layer on hydrogen diffusion (**b**); the trajectories of 10 randomly selected hydrogen molecules in graphene-reinforced systems at 200 K (**c**); and their trajectories at 400 K (**d**) [48]. Copyright 2022 Elsevier.

**Figure 19:**
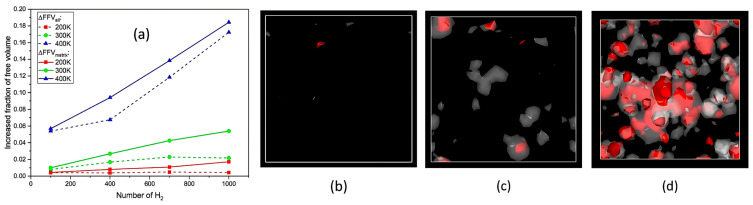
The ΔFFV_matrix_ (solid line) and ΔFFV_all_ (dashed line) in relation to the H_2_ content within systems containing 1000 H_2_ molecules, evaluated with a probe size of 3 Å (**a**). Additionally, the free volume distribution in the simulation cell for the 1000 H_2_ system is displayed at temperatures of 200 K (**b**), 300 K (**c**), and 400 K (**d**), highlighting the part in red that corresponds to the ΔFFV_all_ and the section in white that indicates the ΔFFV_matrix_ [48]. Copyright 2022 Elsevier.

**Figure 20:**
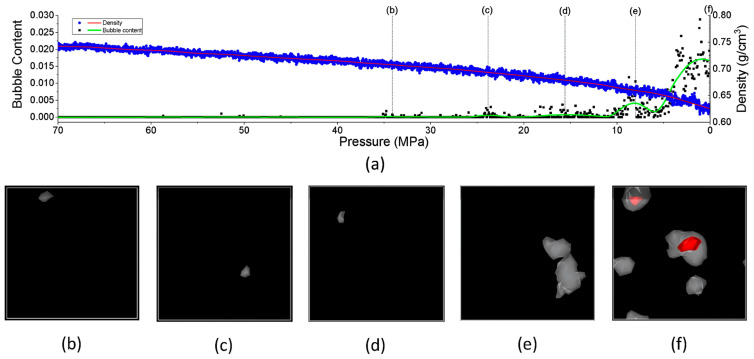
(**a**) The relationship between bubble content and pressure in the PE-1000 hydrogen system, measured with a probe size of 6 Å. Panels (**b**) through (**f**) display the distribution of H_2_ bubbles at various pressures: 34.12 MPa (**b**), 23.83 MPa (**c**), 15.57 MPa (**d**), 8.01 MPa (**e**), and 0.1 MPa (**f**). In these visualizations, the red regions represent free volume, while the white regions indicate free volume where H_2_ has been excluded [48]. Copyright 2022 Elsevier.

- The effect of H_2_ molecules on tensile properties and T_g_: The incorporation of H_2_ into PE significantly affects its tensile properties and T_g_. As the hydrogen content increased, density and tensile modulus clearly decreased. The yield stress initially increased with H_2_ concentrations below 300 molecules but subsequently decreased with higher H_2_ contents, which was attributed to the poor interaction between H_2_ molecules and polyolefin chains. H_2_ molecules do not enhance the mechanical performance of the matrix and act similarly to voids, thereby affecting the tensile properties owing to their mobility and weak interaction with PE. Additionally, the calculated *T*_g_ values for PE decreased with increasing H_2_ content, from 268.0 K for 0 H_2_ molecules to 263.0 K for 500 H_2_ molecules. However, obtaining a *T*_g_ for PE with 1000 H_2_ molecules proved challenging owing to abnormal trends, likely resulting from phase separation, emphasizing the complexity of the influence of hydrogen on the properties of the material.
- Influence of polymer structure on the D: Examining degree of polymerization, side chains, and orientation: The diffusion constant of hydrogen in PE does not show a significant correlation with the degree of polymerization (DP), particularly when the chain length is long, which results in fewer end groups that affect the diffusion rates (Figure 17a). Prior studies have suggested a negative correlation between the diffusion rate and DP, but this research infers that beyond a certain DP, other factors such as the molecular structure and processing conditions (including crystallinity and orientation) play more critical roles. Additionally, the diffusion pathways of H_2_ molecules at different temperatures (200 and 400 K) revealed that at lower temperatures, H_2_ molecules exhibit limited movement mainly within stable free volume regions (Figure 17b), whereas at higher temperatures, their trajectories become more dispersed and irregular, suggesting that a free volume redistribution occurs as the polymer transitions to a rubbery state, which may deviate from the relationship between the diffusion constant and the mean squared displacement established from the Einstein relation (Figure 17c). Figure 17d illustrates the correlation between the orientation of PE chains and the hydrogen diffusion constant. Increased chain orientation led to a reduction in the diffusion constant for hydrogen within the polymer matrix. This phenomenon can be attributed to the denser and more compact structure created by the oriented chains, which restricts the movement of H_2_ molecules compared to a system lacking such orientation. As a result, the compact arrangement of the oriented chains presents a barrier that impedes the free diffusion of hydrogen.
- Influence of additives and reinforcements on hydrogen diffusion in PE: The inclusion of small molecules, specifically C_20_H_42_, significantly enhances the H_2_ diffusion constant in PE systems at various temperatures and pressures (Figure 18a), demonstrating a plasticizing effect that increases chain mobility by creating additional free volume. This trend aligns with previous research conducted on nylon 11 [2]. However, the relationship between diffusion and small molecules becomes less clear in systems operating above T_g_, where other factors may also influence the diffusion process. Figure 18b presents a comparison of the hydrogen diffusion constants between the PE system and graphene/PE composite systems, along with the trajectories of the 10 H_2_ molecules. The presence of the graphene layer leads to an increase in the hydrogen diffusion constant at lower temperatures, whereas at higher temperatures, it decreases. These findings indicate that the diffusion of H_2_ molecules is enhanced at the interface between graphene and PE, which is attributed to the weak van der Waals interactions between the two materials (Figure 18c). However, the presence of graphene can decrease the diffusion constant, especially at higher temperatures (400 K) (Figure 18d), because it may obstruct H_2_ molecules and constrain the mobility of nearby polymer chains. Additionally, the redistribution of free volume around graphene is likely limited. The simulations were conducted without a pressure drop; however, H_2_ is anticipated to diffuse in the direction of the pressure drop, suggesting that to optimize the effectiveness of graphene as a filler, it should be aligned opposite to the pressure drop direction.
- Formation of bubbles during rapid pressure drops: Figure 19 presents the relationship between the changes in free volume (ΔFFV) for different components in a PE system containing 1000 H_2_ molecules. The solid line indicates ΔFFV_matrix_, whereas the dashed line represents ΔFFV_all_. ΔFFV_matrix_ and ΔFFV_all_ increase with the concentration of H_2_ (Figure 19a), suggesting the formation of voids during depressurization. The free volume distribution across simulations at 200, 300, and 400 K is shown in Figure 19b–d, respectively. As the temperature increases, ΔFFV_all_ increases, with the distribution of free volume indicating that H_2_ molecules may aggregate, particularly at higher temperatures. This reinforces the trend that the H_2_ content within the PE matrix significantly impacts the free volume behavior and highlights the dynamic nature of H_2_ diffusion and aggregation under varying thermal conditions. To gain deeper insights into the behavior of the PE/H_2_ system during rapid depressurization, a 10 ns simulation was performed on a system comprising four branchless PE chains with a DP of 2000, containing 1000 H_2_ molecules, at a temperature of 400 K. Gas bubbles were initially detected at approximately 35 MPa but exhibited instability, appearing and disappearing at lower pressures (24, 16, and 8 MPa). At approximately 5 MPa, the bubbles began to expand, with the final sizes reaching 1–2 nm (Figure 20). These findings suggest that high-pressure conditions can lead to the formation of H_2_ nanobubbles in an oversaturated PE matrix, particularly if depressurization occurs too quickly for adequate diffusion to occur. Methods such as SAXS and XRD are recommended to analyze this phenomenon further.

The research encountered several difficulties, primarily related to the intricacies of all-atom MD simulations, which required substantial computational resources and long simulation times to achieve meaningful results regarding hydrogen diffusion in PE. Additionally, accurately modeling the effects of various polymer chain structures, lengths, orientations, and the presence of small molecules or fillers, such as graphene, posed significant challenges in understanding their collective impact on hydrogen behavior in the amorphous region. Among the notable findings, the study established that hydrogen diffusion is markedly affected by the molecular structure of PE, with branched and side-chain configurations facilitating higher diffusion rates by increasing free volume, adversely affecting barrier properties. The research also highlighted that the tensile strength of the PE matrix diminishes in the presence of hydrogen, lowering the glass transition temperature and indicating a reduced material performance under operational conditions. Moreover, the simulations revealed that rapid depressurization could lead to the formation of hydrogen nanobubbles, emphasizing concerns about blistering and material degradation during fast changes in pressure, which are critical insights for the design and application of hydrogen storage systems.

Recently, Ding et al. [49] utilized atomistic MD simulations to investigate the solubility of hydrogen and the decompression behavior of amorphous PE in high-pressure hydrogen environments, focusing on predicting cavitation failure due to the formation of microscopic cavities from dissolved hydrogen gas within the polymer structure. They analyzed how varying pressure, temperature, and stress influence these properties. Their results indicated that rapid decompression induces volumetric expansion of the polymer matrix, significantly increasing the internal free volume. The interaction of hydrogen molecules with polymer chains further enhances this expansion, contributing to an increase in free volume. These molecular mechanisms provide crucial insights into the changes in hydrogen solubility, variations in free volume, and the potential for cavitation under different conditions. To accurately simulate rapid decompression failures, three all-atom amorphous PE models with a density of 0.85 g/cm^3^ were developed, focusing on the amorphous regions where hydrogen is dissolved. Each model comprises 20 PE chains with 250 monomers, resulting in a cell size of approximately 6.5 × 6.5 × 6.5 nm^3^, and employs the CVFF for modeling molecular interactions. MD simulations were conducted using LAMMPS, beginning with an equilibration phase at 250 K and 0 atm for 8 ns to stabilize critical parameters, which confirmed the model’s validity when compared to experimental data. Given the complexities associated with simulating hydrogen permeation, a tailored PE model with varying concentrations of hydrogen was devised. GCMC simulations were utilized to estimate the realistic hydrogen solubility within the polymer at different temperatures and pressures. The PE system was first equilibrated at high pressures ranging from 100 to 1000 atm before being decompressed to 1 atm. The main conclusions drawn from this study are as follows.

**Figure 21:**
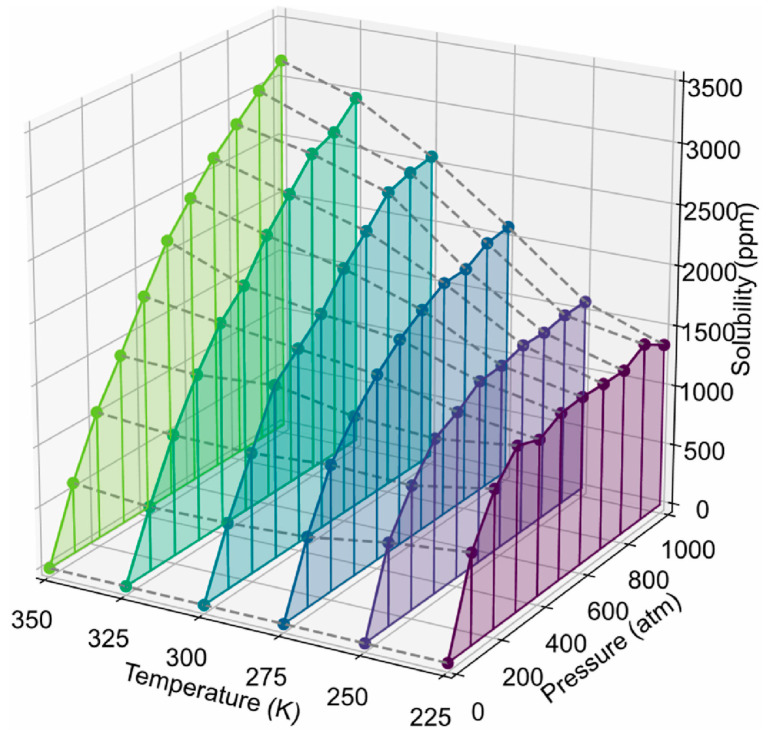
The solubility of H_2_ molecules in amorphous PE as a function of T and P [49]. Copyright 2025 Elsevier.

**Figure 22:**
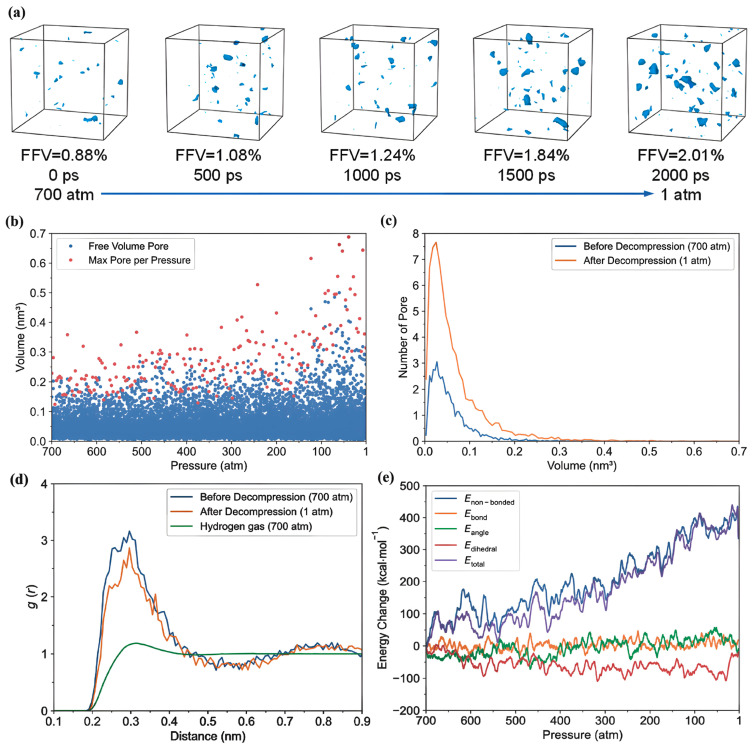
(**a**) Changes in free volume within polyethylene systems containing 155 H_2_ molecules after decompression from 700 atm to 1 atm at a temperature of 300 K, recorded at intervals of 0, 500, 1000, 1500, and 2000 ps; (**b**) a scatter plot depicting the evolution of the size of free volume pores during the decompression process; (**c**) the distribution of free volume pore sizes before and after the decompression event; (**d**) the RDF for H_2_ molecules before and after decompression; and (**e**) variations in energy within the PE system during the rapid decompression phase [49]. Copyright 2025 Elsevier.

**Figure 23:**
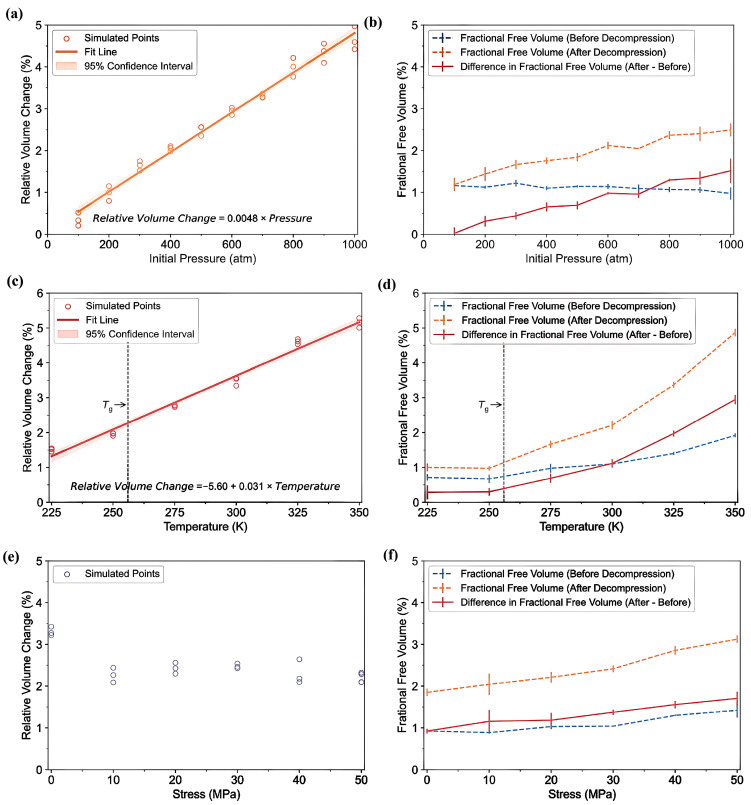
The effect of initial pressure on (**a**) the relative volume changes and (**b**) the FFV in the polyethylene system, both prior to and following decompression. Also, the influence of temperature on (**c**) the relative volume change and (**d**) the FFV in the PE system, evaluated before and after decompression at an initial pressure of 700 atm. Additionally, the impact of tensile stress on (**e**) the relative volume change during decompression and (**f**) the FFV in the PE system, analyzed before and after decompression at a temperature of 300 K and an initial pressure of 700 atm [49]. Copyright 2025 Elsevier.

- Solubility of hydrogen: The analysis of hydrogen solubility in amorphous PE, illustrated in Figure 21, revealed that at 700 atm and 300 K, the solubility reached 2245 ppm. This value is in close agreement with a prior simulation result of 2200 ppm [39] but exceeds the experimental measurement of 1768 ppm [50]. This discrepancy may be attributed to crystalline regions that restrict solubility in actual materials. These findings highlight a clear shift in the solubility behavior. Below the T_g_ of 256 K, the solubility adheres to a Langmuir-type sorption model, where it increases with pressure until saturation is reached. Conversely, above the T_g_, a dual-mode sorption mechanism emerges, revealing a more intricate relationship in which solubility continues to increase with pressure, encompassing elements of Henry’s law and Langmuir sorption as the polymer transitions from a glassy to a rubbery state.
- Energy changes during decompression: Figure 22a depicts the changes in the free volume within PE systems containing 155 H_2_ molecules following decompression from 700 to 1 atm at 300 K. Data were collected at intervals of 0, 500, 1000, 1500, and 2000 ps. During this process, the FFV rose from 0.88% to 2.01%, accompanied by an increase in the number of cavities from 42 to 80, and the average pore size growing from 0.06 to 0.07 nm^3^ at the end of decompression. Figure 22b shows a scatter plot illustrating that as the pressure decreased, the maximum pore size increased, with the majority of pore sizes remaining under 0.2 nm^3^. Additionally, Figure 22c shows a significant increase in the quantity and dimensions of the pores following decompression. In Figure 22d, the RDF reveals that the peak at approximately 0.3 nm for hydrogen clustering remains significant, and the peak height post-decompression is slightly reduced, suggesting an increased dispersion of H_2_ molecules within the PE matrix. The analysis of internal energy shows that energy changes during decompression are predominantly influenced by non-bonded interactions among the polymer chains, particularly as the intermolecular distances increase (Figure 22e). This expansion in intermolecular spacing weakens the cohesive forces within the polymer matrix, establishing favorable conditions for the generation of free volume and consequently, increasing the risk of cavitation damage.
- Factors increasing cavitation risk: Figure 23 highlights the significant effects of the initial pressure, temperature, and tensile stress on the behavior of a PE system during decompression. As shown in Figure 23a, the relative volume change exhibits a linear increase with higher initial pressures, increasing from 0.48% at 100 atm to 4.80% at 1000 atm. In Figure 23b, the FFV exhibits a slight decline before decompression owing to mechanical compression but shows an increase post-decompression, suggesting an elevated risk of cavitation. Furthermore, Figure 23c and d indicate that at an initial pressure of 700 atm, the relative volume changes and FFV increased significantly with increasing temperature. This underscores the fact that temperatures exceeding the T_g_ result in a notable expansion of the free volume, thereby increasing the likelihood of cavitation. Finally, Figure 23e,f demonstrates that although the relative volume changes during decompression remained relatively stable across different levels of tensile stress, the FFV significantly increased with higher applied stress. This indicates that tensile tension promotes the expansion of the free volume, consequently increasing the risk of cavitation within the PE material. The influences of the initial pressure, hydrogen content, temperature, and applied tensile stress notably enhanced the increase in free volume during decompression, further increasing the risk of cavitation.

The key findings of this study highlight the alterations in hydrogen solubility and fluctuations in the FFV of amorphous PE under varying pressures and temperatures, which provide valuable insights into the MD associated with rapid decompression of PE. It was observed that rapid decompression leads to volumetric expansion of PE, increasing the intermolecular distances between polymer chains and resulting in enhanced internal free volume. The solubility of hydrogen within the PE significantly influences this process; as more hydrogen molecules fill the spaces between the chains, they contribute to additional free volume within the matrix. The MD simulations quantitatively assess the impact of initial pressure on the decompression behavior of PE, revealing that higher initial pressures significantly elevate free volume, primarily due to increased hydrogen solubility. Furthermore, the mechanical properties of the polymer are notably affected by temperature; higher temperatures generally lead to reduced mechanical performance, consequently increasing the risk of cavitation [51]. The study demonstrated that at the nanoscale, higher temperatures correlate with a marked rise in free volume during decompression, similar to the effects of increased tensile stress. These observed trends in free volume generation with varying temperature, pressure, and tensile stress correlate well with experimental findings related to cavitation damage, reinforcing the notion that free volume generation is indicative of potential cavity formation at the nanoscale, which can ultimately lead to failure [52].

To mitigate cavitation-induced failure in the polymer liners of hydrogen storage tanks, several strategies have been proposed. One critical approach is to manage hydrogen solubility by designing polymers that limit hydrogen uptake or by controlling the concentration of hydrogen during usage, thus reducing the effect of dissolved hydrogen on free volume expansion during decompression. Additionally, it is essential to lower the initial pressure during decompression, as the analysis indicates that significant increases in FFV occur when pressures surpass 700 atm. Temperature management is another crucial aspect, as elevated temperatures tend to enhance free volume, promoting the formation of cavities, with results showing that temperatures exceeding 300 K significantly increase FFV during decompression. It is also noted that increased temperatures diminish the mechanical strength of the polymer [13], elevating the chances of failure upon decompression. Minimizing tensile stress on the polymer liner is recommended to reduce cavitation risks, as lower stress levels can prevent excessive material deformation and limit the development of large free volumes.

Among the major limitations of this work is the constraint of MD simulations, which restricts the time and spatial scales that can be effectively modeled. Additionally, the study’s focus on only the amorphous regions of PE does not account for crystalline components, which may hinder the applicability of findings to real-world semicrystalline materials. Furthermore, the MD model used does not incorporate specific damage criteria, which limits the capacity to predict material failure onset.

To overcome these limitations, future research could implement coarse-grained MD simulations to incorporate crystalline regions, thus enhancing the understanding of semicrystalline PE behavior under decompression. Expanding the research to include a variety of polymers and composite materials would also improve the generalizability of the findings across different hydrogen storage solutions. Future studies might also establish relevant damage criteria by determining critical thresholds for free volume pore sizes, aiding in the assessment of material degradation and failure mechanisms.

#### 3.1.5. Effect of Nanofillers

Various additives are frequently added to polymers throughout the production process in order to acquire desired qualities. These additives have a major impact on the polymers’ gas barrier properties. They include fillers and plasticizers, including crosslinking agents. By modifying the degree of cross-linking amongst molecular chains, crosslinking agents can change the distance between them and affect the penetration of gas molecules. Greater levels of crosslinking limit molecular chain mobility, which makes it harder for bigger penetrants to permeate the material.

Wu et al. [11] improved the polymer liner of type IV hydrogen storage bottles to increase their hydrogen storage capacity. Their study used molecular dynamics simulations to investigate the adsorption and diffusion of helium in a polyamide 6 (PA6) composite system filled with modified montmorillonite (OMMT). MMT is an aluminosilicate mineral with a strong ion-exchange capacity. MMT cells were created with lattice dimensions a = 5.23 Å, b = 9.06 Å, and c = 12.5 Å using Material Studio. A supercell of size 4a × 2b × 1c was formed by adding atoms based on spatial and cation coordinates. The SPC/E model treated water as a rigid molecule, using six Na^+^ interlayer cations, later replaced with octadecyl trimethylammonium chloride for PA6 compatibility. Composite models were created with 100 PA6 molecules, integrating modified MMT with OMMT mass fractions of 3% to 7%. Unit cell sizes for these fractions ranged from 75.51 Å to 56.47 Å. Geometric optimization was performed with 50,000 iterations and cyclic annealing at 300 K to 500 K. MD simulations in an NPT ensemble used the UFF force field for 1000 ps at 298 K and 0.1 MPa, controlling temperature with the Andersen method and pressure with the Berendsen method. Helium solubility coefficients were determined through adsorption isotherms across a pressure range from 0.01 kPa to 10,000 kPa. Simulations at 0.1 MPa, 41.6 MPa, 52 MPa, and 60 MPa were conducted for hydrogen storage tanks, assessing helium permeation and analyzing the MSD curve to calculate the diffusion coefficient. The main results indicated that the optimal filler content was 5% OMMT, which produced the best barrier performance with a permeability coefficient lower than 2 × 10^−13^ cm^3^·cm/(cm^2^·s·Pa) at 328 K (Table 1). This optimal formulation effectively restricted gas diffusion, demonstrating a significant reduction in solubility (60% lower), diffusion coefficient (15% lower), and permeability (60% lower) compared to 3% OMMT content. The study concluded that increasing the OMMT content beyond 5% led to increased permeability due to the creation of additional diffusion pathways, which counteracted the intended barrier enhancement.

Significant challenges in the study included the limitations of simulating helium, a substitute for hydrogen, complicating the accurate reflection of the gas barrier properties, especially under high temperatures (288 K and 328 K) and varying pressures (up to 60 MPa). The complexity of molecular interactions within the polymer matrix and the difficulty in modeling the diffusion behavior under these conditions posed additional hurdles. Furthermore, the relationship between free volume and gas permeability was crucial, as larger free volumes correlated with higher diffusion coefficients, thus influencing the barrier performance. Overall, the findings emphasized the importance of optimizing filler content and understanding polymer structure to improve barrier materials for hydrogen storage, providing valuable insights for future research in polymer composites and gas storage materials.

**Figure 24:**
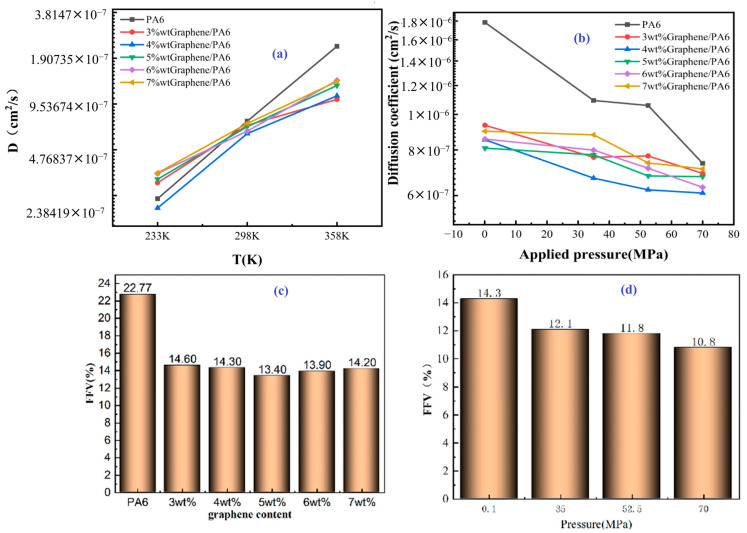
(**a**) Diffusion coefficients of six systems at various temperatures; (**b**) diffusion coefficients of six systems at 298 K under varying pressure conditions; (**c**) FFV for different filler contents at 298 K and 0.1 MPa; (**d**) FFV of the 4% graphene/PA6 composite under four distinct pressure conditions [9]. Copyright 2024 MDPI.

Li et al. [9] employed MD simulations to investigate hydrogen adsorption and diffusion in graphene-filled PA6 systems intended for Type IV hydrogen storage cylinders. The study constructed polymer liners using amorphous unit cells, wherein single-chain structures of PA6 were formed with 60 repeated units. To analyze hydrogen diffusion, five different systems were modeled, each containing 20 H_2_ molecules paired with identical PA6 chains of eight repeated units. The graphene mass fraction was adjusted by incrementally increasing the number of graphene sheets to achieve mass fractions ranging from 3 wt% to 7 wt%. The unit cell dimensions for these varying mass fractions were consistent, spanning from approximately 44.06 Å to 44.40 Å. To enhance the polymer structure, geometric optimization was conducted using the Smart method, followed by a cyclic annealing process that ranged from 300 K to 600 K over 30 cycles. The finalized models underwent MD simulations utilizing the COMPASS II force field in an NVT ensemble at 298 K for a duration of 1 ns. Temperature and pressure were maintained throughout the simulations using the Andersen and Berendsen control methods, respectively. Non-bonded interactions were assessed via the group-based method, and the integration of the equations of motion was executed with a time step of 1 fs. The key findings regarding the permeability of the modified material indicated the following:

- The diffusion coefficients of six systems at different temperatures (233 K, 298 K, and 358 K) show an increase with temperature, as illustrated in Figure 24a. At 358 K and 70 MPa, pure PA6 has the highest diffusion coefficient of 2.28 × 10^−6^ cm^2^/s, attributed to enhanced molecular mobility with rising kinetic energy [26]. A prior study by Dong et al. [26] reported a similar coefficient of 2.36 × 10^−6^ cm^2^/s for PA6 at 358 K and 87.5 MPa, with a 10.6% error margin deemed acceptable for gas diffusion measurements. The diffusion coefficients for graphene/PA6 composites also increase with temperature; for instance, the 3 wt% composite at 358 K shows a 48% increase compared to 298 K. Higher graphene concentrations (4 wt% to 7 wt%) lead to increases in diffusion coefficients by approximately 77%, 138%, 115%, and 89%, highlighting the considerable effect of temperature on diffusion properties, aligning with earlier findings [53].
- The diffusion coefficients of hydrogen decrease linearly with increasing pressure for all tested materials, with pure PA6 exhibiting the highest coefficients and the 4 wt% graphene/PA6 composite showing the lowest (Figure 24b). Permeability coefficients also decline as pressure rises. At 0.1 MPa, the 5 wt% graphene/PA6 composite has the lowest permeability at 2.44 × 10^−13^ cm^3^·cm/(cm^2^·s·Pa), while the 4 wt% composite records the lowest permeability values (2.15 × 10^−13^ to 1.96 × 10^−13^ cm^3^·cm/(cm^2^·s·Pa)) above 35 MPa. This trend aligns with findings that increased pressure compresses the polymer and reduces free volume, hindering H_2_ diffusion. Additionally, higher pressures lead to increased polymer crystallinity, further restricting hydrogen diffusion.
- The FFV directly affects the diffusion coefficients of materials, with a larger FFV resulting in higher diffusion coefficients (Figure 24c,d). In Gr/PA6 composite systems, hydrogen diffusion occurs through a “vibration + leap” mechanism. The 3 wt% Gr/PA6 composite shows significant vibrational movements of hydrogen, whereas the 7 wt% composite enables both substantial forward and backward leaps of H_2_ molecules. Furthermore, increased pressure in the 4 wt% Gr/PA6 system leads to longer leap distances and higher leap frequencies for hydrogen.

### 3.2. Multi-Scale Modeling of Composite Hydrogen Storage Tanks

**Figure 25:**
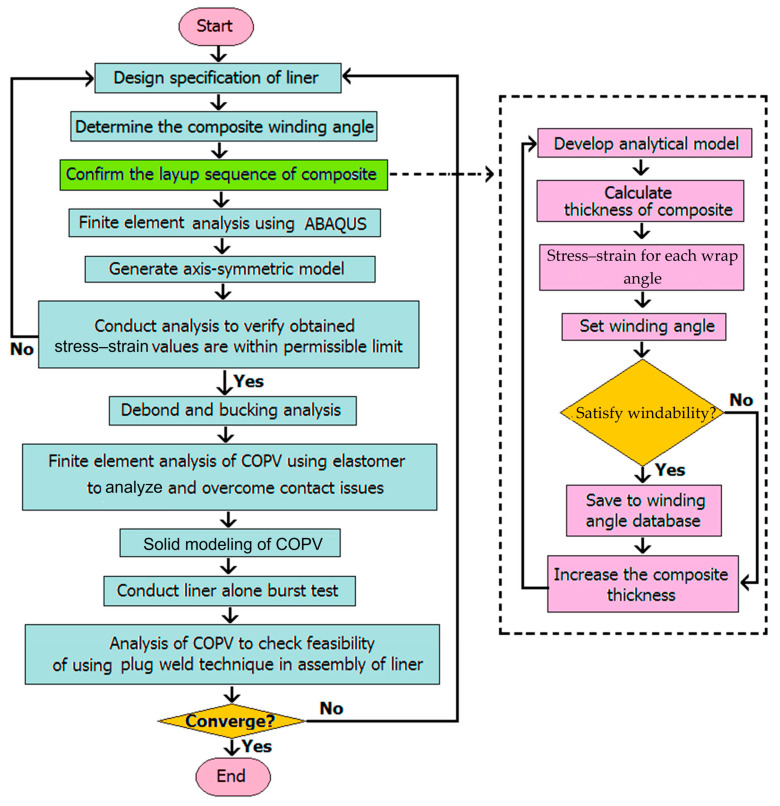
Flowchart illustrating the finite element modeling process for a composite tank [62]. Copyright 2023 MDPI.

**Figure 26:**
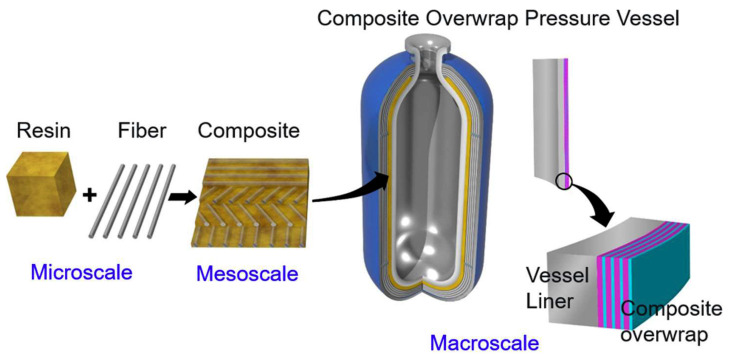
Diagram depicting the multiscale mechanistic method for analyzing hydrogen storage composite pressure tanks [62]. Copyright 2021 Elsevier.

Multi-scale modeling of composite hydrogen storage vessels entails the application of mathematical models and simulation tools to evaluate the performance of these vessels across various scales (as shown in Figure 25). This approach may involve simulating the behavior of individual materials and components at the microscale, as well as predicting the overall performance of the vessel at the macroscale [54]. Such modeling is essential for comprehending the intricate interactions within composite hydrogen storage vessels and determining the factors that affect their performance. Additionally, it aids in optimizing the design and fabrication of these vessels by pinpointing the materials and methods that yield the best results. For multi-scale modeling, researchers often utilize software like ABAQUS or ANSYS (refer to Figure 26), along with specialized tools tailored for this type of analysis [54]. They may also draw upon experimental data from tests and simulations to validate and enhance their models. Ultimately, the objective of multi-scale modeling is to gain a deeper understanding of the behavior of composite hydrogen storage vessels and to enhance their overall performance.

**Figure 27:**
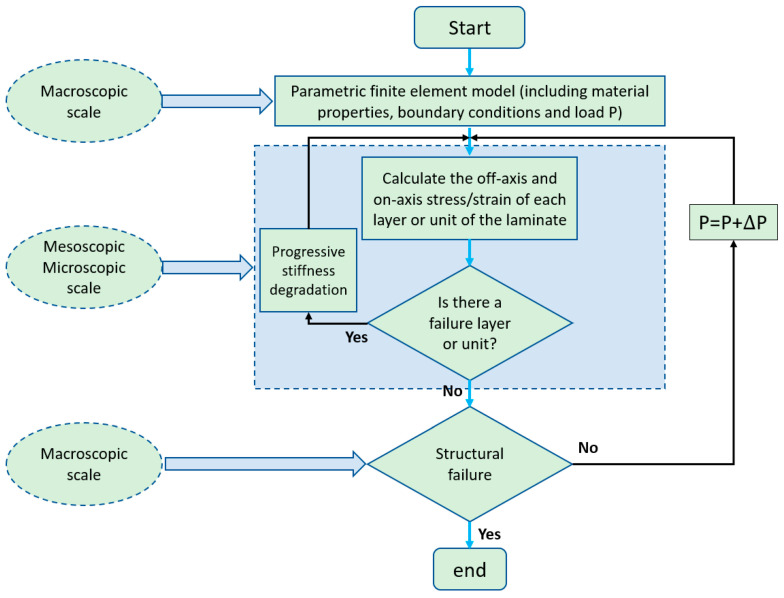
Diagram illustrating the progressive failure analysis of composite tanks [63]. Copyright 2023 MDPI.

Recently, advancements in failure criteria have propelled the evolution of failure analysis, shedding light on the interactions between the microscopic and macroscopic elements of composite materials. The behavior following initial failure in composite laminate structures is modeled through the material property degradation method, particularly using a combination of CDM and commercial finite element analysis techniques. The development of multi-scale failure analysis has progressed through innovative finite element methods, including cohesive element (CE) and RVE approaches. The following section provides a summary of the progressive failure analysis applied to composite tanks using the finite element method as a foundation for further analysis [55]. Figure 27 illustrates the flowchart of this progressive failure analysis process, which typically encompasses (I) stress analysis, (II) failure evaluation, (III) material degradation, and (IV) burst pressure assessment.

**Figure 28:**
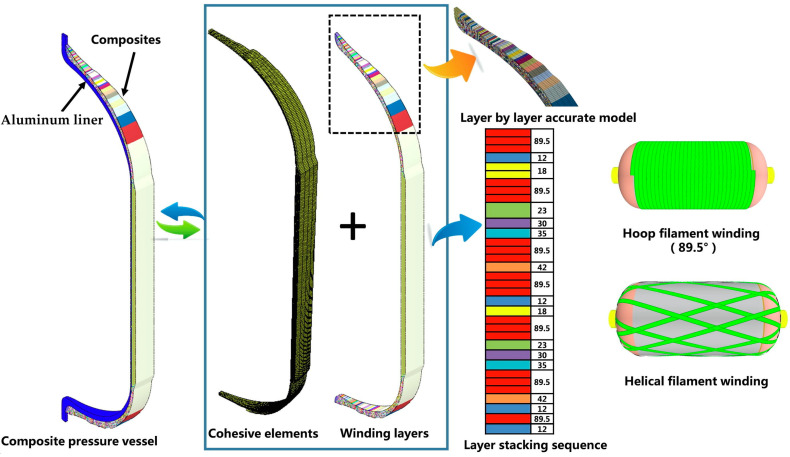
FEA models of composite pressure tanks [56]. Copyright 2021 Elsevier.

Recently, Lin et al. [56] introduced a method for analyzing progressive failure in Type III composite pressure vessels rated at 35 MPa during hydraulic burst tests. They employed RVEs to calculate the degraded elastic parameters of the composite, accounting for fiber and matrix failure modes, using FEA to derive macroscopic stiffness degradation metrics. The FEA model of the composite pressure vessel was created using Abaqus 6.14, employing a 1/36 periodic symmetric model to optimize computational resources (Figure 28). The Puck failure criterion was utilized, and effective elastic parameters from RVEs were implemented through the UDSLFD subroutine to simulate multiscale damage progression during testing. Combining this with acoustic emission monitoring, microscopic analysis, and burst tests, they examined the vessels’ burst pressure and failure mechanisms. The findings demonstrated that meso-macroscopic finite element models accurately predicted damage progression, including matrix cracking and fiber fracture, under internal pressure. The predicted burst pressure showed only a 5.4% deviation from the average experimental results, and the predicted failure location closely matched observed test sites. This methodology offers valuable insights for the design and optimization of composite pressure vessels.

**Figure 29:**
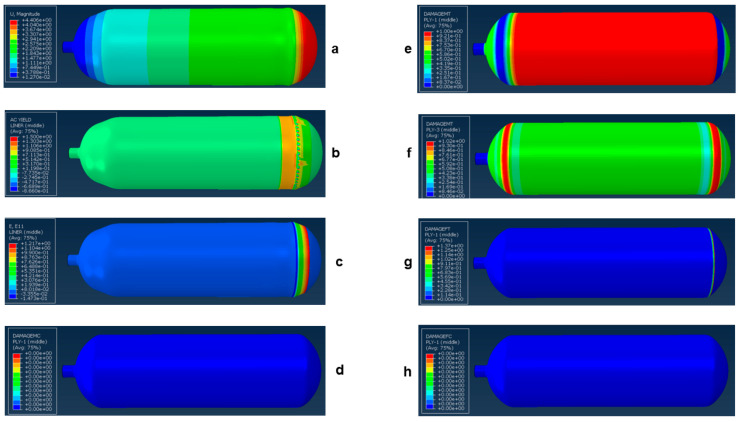
Damaged tank obtained using Hashin criterion and the vessel response using conventional shell elements model at failure for a stacking of 24 plies: (**a**) magnitude of the displacement, (**b**) yield response in the polymeric liner, (**c**) axial strain in the liner, (**d**) compression damage of the matrix in the first ply, (**e**) tensile damage of the matrix in the first ply, (**f**) damage of the matrix in tension at the third ply, (**g**) damage of the fiber in tension at the first ply, (**h**) damage of the fiber in compression at the first ply [64]. Copyright 2024 MDPI.

Son et al. [57] utilized finite element analysis to simulate the autofrettage process of a type III hydrogen pressure vessel. This procedure involves subjecting the vessel to internal pressure exceeding its elastic limit, resulting in plastic deformation of the inner layers. Upon pressure release, the outer layers compress the inner layers, creating beneficial residual compressive stresses that enhance the vessel’s capability to endure high internal pressures, ultimately improving its fatigue life and resistance to stress corrosion cracking. The study examines stress distribution and the residual stresses generated to identify the optimal autofrettage pressure. Additionally, it forecasts failure under minimum burst pressure by applying various failure criteria for anisotropic composites, providing valuable insights into the autofrettage process for type III hydrogen pressure vessels. Wu et al. [58] performed stress and damage analyses on composite overwrapped pressure vessels with an aluminum liner through numerical simulations utilizing a progressive damage model. They investigated the initiation and distribution of different types of damage, along with their effects on burst pressure and autofrettage pressure. Tsai et al. [59] introduced a strength theory for anisotropic materials, employing a function of two strength tensors while addressing coordinate transformations, independent interaction terms, and symmetry in materials. Furthermore, Park et al. [60] examined crack behavior in type III high-pressure hydrogen vessels using the ply modeling method and extended finite element method (FEM). Failure criteria based on maximum principal stress and displacement were applied to analyze cracks in the carbon fiber-reinforced plastic layer, leading to the identification of weak points and providing critical insights to enhance the safety of high-pressure hydrogen vessels, as illustrated in Figure 29.

**Figure 30:**
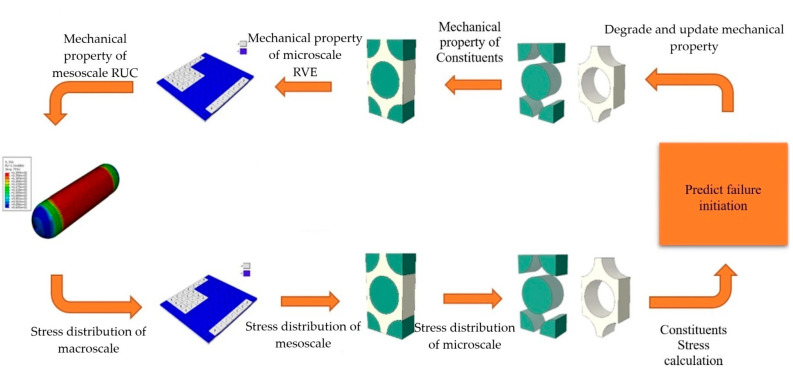
Summary of the recursive multi-scale modeling approach [61]. Copyright 2022 Springer.

This approach requires extensive mechanical testing for lamina strength data, particularly for temperature-sensitive materials. However, the micro-meso-macro approach proves more efficient for complex laminated structures, such as filament-wound pressure vessels, as it better reflects the actual mechanical properties compared to data from flat specimens. Despite significant advancements in continuum damage mechanics for laminated composites, its application in pressure vessels remains limited. Damage evolution in transverse matrix cracking is modeled within this framework, and fiber rupture during loading is predicted using a micromechanical criterion. Furthermore, a new recursive multi-scale model by Rafiee et al. [61] has been developed to predict burst pressure, addressing imperfections in fiber arrangement throughout the filament winding process (Figure 30).

### 3.3. Artificial Intelligence for Hydrogen Storage Vessels

**Figure 31:**
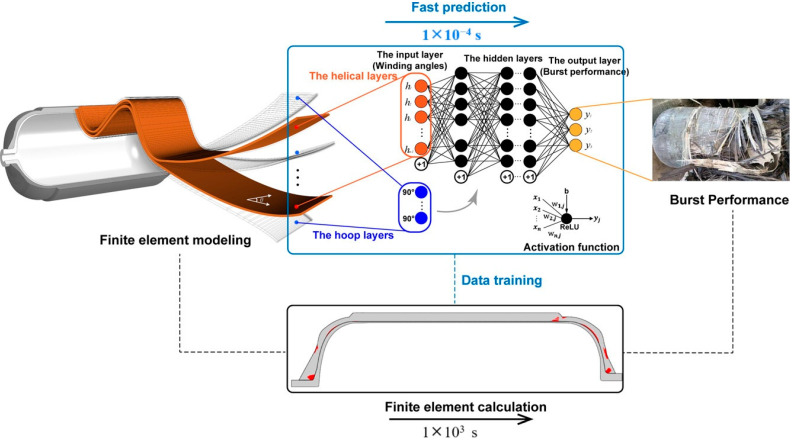
ML combined with FEA for optimizing type IV hydrogen storage vessel design [70]. Copyright 2023 Elsevier.

**Figure 32:**
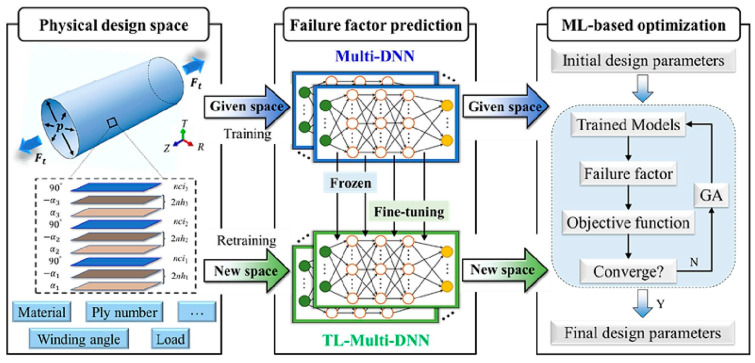
Flowchart illustrating the prediction of failure factors (R) for CPVs using various design parameters and an optimization scheme that integrates the trained models with genetic algorithms (GA) [71]. Copyright 2024 Elsevier.

Artificial intelligence (AI) plays a vital role in improving the design and functionality of hydrogen storage vessels. The incorporation of AI into the development and optimization of composite storage vessels not only boosts performance but also enhances safety and sustainability. By automating numerous labor-intensive and time-consuming tasks involved in design and analysis, AI greatly speeds up the process of creating efficient and effective composite storage solutions [65,66,67]. Machine learning (ML) is a powerful computational approach that utilizes algorithms and statistical models to analyze and interpret complex data, enabling systems to learn from experience and make predictions or decisions without being explicitly programmed [68,69]. In the context of engineering and material science, ML has gained significant attention for its ability to enhance the design and optimization of composite materials, including the increasingly complex geometries of structures like type IV hydrogen storage vessels. By analyzing large datasets generated from FEA, ML can uncover patterns and relationships that may not be readily apparent, making it a valuable tool for predicting material behavior, optimizing structural performance, and reducing computational costs associated with traditional numerical methods. This capability is particularly crucial in applications involving intricate geometrical details and varying material properties, allowing for rapid assessments of potential design configurations and performance outcomes [70]. A variety of AI models have been utilized in the literature for the design of hydrogen storage vessels, showcasing their growing significance in this field. Recently, Li et al. [70] established a framework that integrates ML with FEA to optimize the design of thin-layered composites in type IV hydrogen storage vessels (Figure 31). By coupling an ANN with FE models, they effectively optimized the winding parameters and dome geometries, resulting in a significant reduction in computational costs and a prediction error of less than 2% in the damage state function. This optimization led to an increase in burst pressure from 145 MPa to 157.74 MPa, corroborated by experimental validation of the improved design. Wang et al. [71] investigated the prediction of failures and optimization processes in composite pressure vessels by integrating FEM simulations with ML. Their study focused on predicting the failure factors (R) of CPVs through a deep neural network (multi-DNN) that incorporates transfer learning (Figure 32). This method enhances the accuracy of predictions for CPVs with new design parameters, while facilitating efficient optimization via genetic algorithms. As a result, they achieved practical estimations and optimizations of CPVs, all while lowering computational costs. Additionally, Kadri et al. [72] utilized machine learning to examine the degradation of composite polymers used in high-pressure hydrogen vessels, seeking to forecast material performance and longevity in operational settings. Additionally, artificial intelligence has been leveraged to improve the modeling and design of composite pressure vessels. Hong et al. [73] employed deep transfer learning techniques to predict the behavior of these vessels with greater accuracy and efficiency. Islam et al. [74] showcased the potential of AI in detecting possible vessel failures, achieving an impressive 94.67% accuracy in classifying cracks using acoustic emission signals and deep learning methodologies. Beyond predictive capabilities, AI also provides useful tools for assessing probabilistic behavior and reliability. Azizian and Almeida [75] merged simulations with artificial neural networks to forecast the performance of composite tubes under varying loading conditions. Furthermore, AI’s ability to develop “digital twins” for vessels, as demonstrated by Hopmann et al. [76], allows for the prediction of future behaviors based on manufacturing processes. Qarssis et al. [77] explored the use of machine learning for analyzing the mechanical behavior of filament-wound, thin-composite hydrogen storage tanks under internal pressure. Their innovative approach combines classical laminate theory, extensive parametric analysis, and machine learning techniques, advancing composite tank design and potentially aiding other complex structural systems. They conducted a comprehensive parametric study on various design factors, such as internal pressure and layer orientation, using Pearson’s correlation analysis to identify significant influences on tank performance. Five machine learning models were assessed, with Extreme Gradient Boosting (XGBoost) demonstrating superior predictive accuracy (99%) for stress prediction in the x-direction, outperforming Random Forest. A user-friendly GUI application was also developed to facilitate real-time simulation of mechanical behavior by adjusting design variables, streamlining the design process, and enhancing efficiency and reliability.

## 4. Challenges and Future Perspectives

This review identified significant challenges associated with hydrogen gas permeation in polymer liners for type IV hydrogen storage cylinders. Common materials such as HDPE and PA exhibit high permeability, leading to issues such as hydrogen blistering and structural failure. The accurate measurement of hydrogen gas permeability under varying conditions complicates compliance with international standards, highlighting the need for improved testing methods that ensure reliability and consistency. Future research should prioritize the development of advanced polymer materials with lower permeability and enhance methodological approaches to enhance hydrogen storage safety. Additionally, this review highlighted the complexities of polymer behavior under high-pressure hydrogen, particularly the effects of rapid decompression on material integrity. The limited literature on the joint impact of pressure and temperature contributes to inconsistencies in the experimental data, emphasizing the necessity to investigate the aging phenomena affecting polymer properties further. Future studies should aim to create experimental setups that realistically simulate operational conditions to shed light on the damage mechanisms, thereby improving the safety of hydrogen storage and distribution. Research on the behavior of PE in high-pressure environments faces challenges such as insufficient experimental data and discrepancies between MD simulations and real-world findings. Future efforts should focus on refining the understanding of polymer behavior under various stress conditions and enhancing the validity of MD simulations to guide the development of materials optimized for high-pressure hydrogen applications. Moreover, MD simulations of hydrogen diffusion in amorphous PE struggle with accuracy because of the influence of the molecular structure, orientation, crystallinity, and processing conditions on the gas solubility and diffusion behavior. Furthermore, the small size of H_2_ molecules complicates the experimental validation of diffusion constants, affecting reproducibility and safety. Future research should aim to improve the barrier properties against hydrogen diffusion through optimized bonding and orientation of nanofillers such as graphene. In situ studies using techniques such as SAXS and wide-angle XRD are recommended to explore bubble formation during rapid depressurization and alternative polymer formulations that address processing issues related to viscosity and molecular weight. Traditionally, type IV cylinders utilize a thermoplastic liner overwrapped with a fiber-reinforced thermoset composite; however, this hybrid configuration suffers from poor fatigue performance at the liner–overwrap interface owing to bonding challenges. Adopting type V construction, in which the liner and overwrap are thermoplastic, could mitigate this issue. An illustrative example is the UK consortium DuraStor, which is developing a lighter, more cost-effective, and all-thermoplastic composite storage vessel by combining a POM liner with a carbon fiber/POM composite overwrap. Similarly, the French initiative HYPE created compressed hydrogen vessels wrapped in a PA matrix/CF composite, branded as Carbostamp [78].

## 5. Conclusions and Research Gaps

The development of high-performance polymer liners is essential for safe hydrogen storage in type IV tanks. Significant progress has been made in material selection, fabrication, and molecular modeling to enhance hydrogen gas barrier performance, with polymers such as HDPE and PA6 noted for their strength and gas barrier properties. The use of nanofillers, particularly graphene and modified montmorillonite clay, effectively reduces hydrogen gas permeability while maintaining flexibility. MD simulations are crucial for understanding permeation mechanisms; however, the research on polymer aging has a notable gap due to environmental factors. Current findings show that hydrogen solubility and permeability in amorphous PE increase with temperature, with a minimal pressure impact. However, experimental validation is still required, particularly for hydrogen transport in PE pipelines. The hydrogen gas permeability of HDPE and EVOH is mostly temperature-dependent, with HDPE showing increased permeability owing to its weaker hydrogen bonding capabilities. However, the long-term effects of hydrogen exposure on polymers, particularly with respect to aging and mechanical stress, are not yet well understood. Hydrogen exposure decreases the tensile properties of amorphous PE, increasing the free volume and hydrogen diffusion rates, whereas polymer branching may compromise the barrier properties. Future investigations should explore the long-term performance of polymer materials under hydrogen exposure and various conditions. This review showed that PA6 modified with montmorillonite clay exhibits optimal barrier performance against helium at a 5 wt% filler content. However, its long-term durability under pressure and environmental conditions remains unclear. Additionally, simulations indicated that 5 wt% graphene filler in PA6 significantly reduces the hydrogen gas permeability. Further research is required to explore the diffusion coefficients and validate the practical applications of these modified materials in hydrogen storage systems.

## Figures and Tables

**Table 1:** Solubility (*S*), diffusion coefficient (*D*) and permeation coefficient (*P*) of helium in different systems and conditions [11]. Copyright 2023 MDPI.

Matrices	S (cm^3^/cm^3^.Pa).10^−7^	D (cm^2^/s).10^−6^	P (cm^3^.cm/cm^2^.s.Pa).10^−13^
288 K and 0.1 MPa			
PA6/3%OMMT	2.23	1.72	5.56
PA6/4%OMMT	1.93	1.65	3.18
PA6/5%OMMT	1.26	1.48	1.85
PA6/6%OMMT	1.55	1.58	2.54
PA6/7%OMMT	1.60	2.21	3.54
328 K			
PA6/3%OMMT	2.58	5.38	10.39
PA6/4%OMMT	1.51	3.58	5.41
PA6/5%OMMT	1.02	2.66	2.71
PA6/6%OMMT	1.17	3.15	3.63
PA6/7%OMMT	1.32	4.82	6.43
5%PA6/OMMT at 288 K			
0.1 MPa	1.26	1.48	1.86
41.6 MPa		1.38	1.74
52 MPa		3.41	4.30
60 MPa		4.09	5.15

## Data Availability

Data are contained within the article. Further inquiries can be directed to the corresponding author.

## Abbreviations

**PE**	Polyethylene
**FFV**	Fractional free volume
***T*_g_**	Glass transition temperature
**MD**	Molecular dynamics simulation
**P**	Pressure
**T**	Temperature
**HDPE**	High-density polyethylene
**S**	Solubility coefficient
**MC**	Monte Carlo simulation
**MMT**	Montmorillonite
**MSD**	Mean square displacement
**PA6**	Polyamide 6
**D**	Diffusion coefficient
**PEEK**	Polyether ether ketone
**EVOH**	Ethylene-vinyl alcohol copolymer
**PFSA**	Perfluorosulfonic acid polymer
**CFRP**	Carbon fiber reinforced plastic
**RDF**	Radial distribution functions
**P**	Permeability coefficient
**MS**	Materials Studio
**OMMT**	Modified montmorillonite
**GCMC**	Grand Canonical Monte Carlo
**DP**	Degree of polymerization
**RGD**	Rapid gas decompression
**HE**	Hydrogen embrittlement
**LIC**	Lamellar inorganic components
**CNC**	Computer numerical control
**PPS**	Polyphenylene sulfide
**POM**	Polyoxymethylene
**UTM**	Universal testing machine
**T_m_**	Melting temperature
**T_c_**	Crystallization temperature
**SAXS**	Small-angle X-ray scattering
**CDM**	Continuum damage mechanics
**RVE**	Representative volume element
**ANN**	Artificial neural network
**FEM**	Finite element method
**CPVs**	Composite pressure vessels
**CVFF**	Consistent valence forcefield
